# Protocol to create isogenic disease models from adult stem cell-derived organoids using next-generation CRISPR tools

**DOI:** 10.1016/j.xpro.2024.103189

**Published:** 2024-07-12

**Authors:** Martina Celotti, Lucca L.M. Derks, Johan van Es, Ruben van Boxtel, Hans Clevers, Maarten H. Geurts

**Affiliations:** 1 Hubrecht Institute, Royal Netherlands Academy of Arts and Sciences (KNAW) and University Medical Center Utrecht, 3584 CT Utrecht, the Netherlands; 2 Oncode Institute, 3521 AL Utrecht, the Netherlands; 3 Princess Maxima Center for Pediatric Oncology, 3584 CS Utrecht, the Netherlands

**Keywords:** Cancer, Genetics, CRISPR, Stem Cells

## Abstract

Isogenic disease models, such as genetically engineered organoids, provide insight into the impact of genetic variants on organ function. Here, we present a protocol to create isogenic disease models from adult stem cell-derived organoids using next-generation CRISPR tools. We describe steps for single guide RNA (sgRNA) design and cloning, electroporation, and selecting electroporated cells. We then detail procedures for clonal line generation. Next-generation CRISPR tools do not require double-stranded break (DSB) induction for their function, thus simplifying *in vitro* disease model generation.

For complete details on the use and execution of this protocol, please refer to Geurts et al.^1^^,^^2^

## Highlights

• Base- and prime editor guide RNA design for isogenic organoid line generation
• Electroporation-based transfection of CRISPR tools into organoids
• Experimental setup to select for CRISPR-engineered organoids
• Clonal isogenic line generation and Sanger-based validation of genome editing

## Abstract (editor highlights)

Publisher’s note: Undertaking any experimental protocol requires adherence to local institutional guidelines for laboratory safety and ethics.

## Graphical abstract

**Figure:**
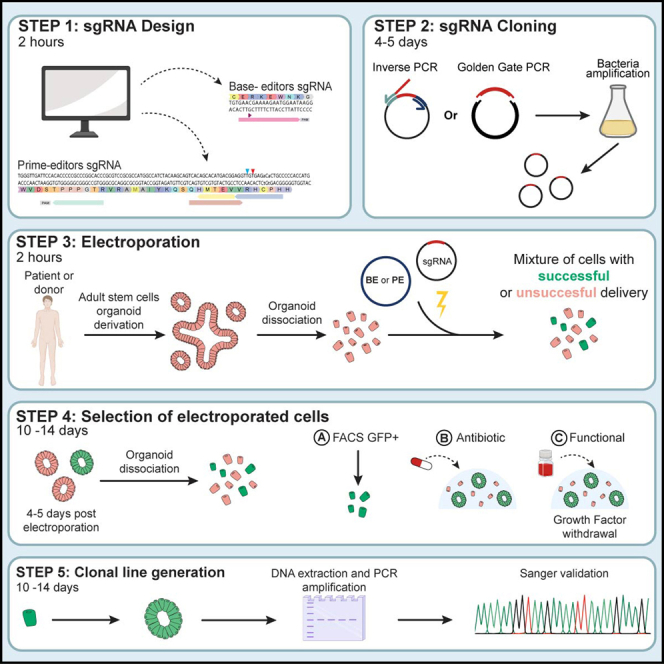

## Before you begin

To accurately assess the impact of genetic variants on both homeostasis and diseases, it is essential to establish precise disease models within a laboratory context. Two-dimensional (2D) cultures have historically served as experimental platforms for disease investigation. However, their ability to faithfully mimic native organ structures is limited.^3^^,^^4^ Murine models offer an alternative avenue for studying the effect of mutations at a tissue scale *in vivo*. Nevertheless, generating a mouse line is time-consuming and mutations that cause diseases in humans do not always result in the same disease phenotype in mice.^5^

With the advent of three-dimensional (3D) adult stem cell (ASC) organoid cultures, there is now a promising middle ground between *in vivo* and *in vitro* studies.^6^ Organoids exhibit a high degree of structural complexity and cellular organization, resembling native tissue architecture more closely than 2D cell cultures. This allows for more accurate recapitulation of organ-specific functions and disease processes.^7^ Additionally, organoids can be derived from both healthy (i.e., wild-type) organs, but also from patients suffering from Mendelian disorders such as cystic fibrosis or directly from tumor material. This enables personalized disease modeling and drug screening.^8^ Thus, ASC organoids provide researchers with more physiologically relevant models for investigating the impact of genetic variants on disease pathogenesis and therapeutic responses.

Creating isogenic disease models that differ only in the DNA mutation of interest is crucial for accurate genetic variant assessment. Clustered Regularly Interspaced Short Palindromic Repeats (CRISPR)-Cas9 gene editing has revolutionized this process.^9^ Conventional CRISPR-Cas9 methods induce double-stranded breaks (DSBs) that are predominantly repaired by error-prone non-homologous end-joining (NHEJ).^10^ While NHEJ facilitates gene knock-out (KO), many diseases arise from point mutations, requiring homology-directed repair (HDR). However, HDR is often less efficient due to the cell’s preference for NHEJ, leading to indel formation rather than desired mutations. Additionally, DSBs repair can cause larger chromosomal rearrangements such as chromothripsis.^11^ Novel CRISPR-based technologies have been developed to address limitations associated with DSBs induction. Next-generation CRISPR tools, such as base editors, use enzymes like cytidine or adenine deaminases fused to Cas9 to introduce single nucleotide variants (SNVs) without DSBs.^12^^,^^13^ Prime editors, combining nickase-Cas9 (nCas9) with a reverse transcriptase, further expand DSBs-free genome engineering, allowing for the introduction of all point mutations and small insertions and deletions.^14^

CRISPR-Cas9-mediated genome engineering has extensively been used to model or repair mutations in ASC organoids.^1^ Here, we detail the pipeline for using DSBs-free genome engineering in ASC organoids, specifically in intestinal, endometrial and hepatocyte organoids.^2^^,^^15^^,^^16^ These protocols are applicable in ASC derived organoids from both murine and human sources and can be derived from various organs.^17^ We describe the complete process of isogenic ASC organoid generation, from choosing the editing technique and designing sgRNAs to delivery into organoids and analysis of edited clones.

### Institutional permissions

All procedures outlined in this protocol were carried out according with all relevant ethical regulations regarding research involving human participants, were approved by the UMC Utrecht (Utrecht, the Netherlands) ethical committee and were in accordance with the Declaration of Helsinki and according to Dutch law. Before any work with organoids derived from primary material is started, it is essential that institute and ethical approval is ensured and that subsequent experiments are following all relevant regulatory standards.

### Growth factors stock preparation

**Timing: 30 min**

**Table:** 

Reagent	Final stock concentration	Amount	Volume
Nicotinamide	1 M	12.2 g	100 mL PBS
N-Acetyl-L-cysteine	500 mM	4 g	49 mL Nuclease free water
SB202190 (p38 inhibitor)	30 mM	25 mg	2.26 mL DMSO
EGF	500 μg/mL	1 mg	2 mL 0.1% BSA in PBS
A83-01 (ALK4/5/7 inhibitor)	5 mM	50 mg	23.7 mL DMSO
Prostaglandin E2	10 mM	100 mg	28.37 mL DMSO
Y-27632 dihydrochloride (Rho-kinase inhibitor)	10 mM	100 mg	31.2 mL Nuclease free water
FGF10	100 μg/mL	100 μg	1 mL 0.1% BSA in PBS
FGF7	100 μg/mL	100 μg	1 mL 0.1% BSA in PBS
HGF	100 μg/mL	100 μg	1 mL 0.1% BSA in PBS
TGF-α	100 μg/mL	100 μg	1 mL 0.1% BSA in PBS
CHIR99021	3 mM	1 mg	In 715 μL DMSO
gastrin	0.1 mM	1 mg	In 4.8 mL PBS

Store at −20°C up to 2 months. Prepare aliquots to avoid repeated freeze/thaw cycles.

**Table:** Other reagents preparation

Reagent	Final stock concentration	Amount	Volume
DAPI	2 mg/mL	10 mg	5 mL Nuclease free water

Store at 4°C up to three months.

### Strategy determination step 1: Picking the right genome editing tool

**Timing: 30 min**

Prior to sgRNA design it is important to pick the most suitable DSBs-free genome engineering technology for your mutation of interest. As there are less variables in base editing versus prime editing, efficiencies of target mutation induction are, in our hands, more predictable without extensive optimizations.^18^

**Figure 1:**
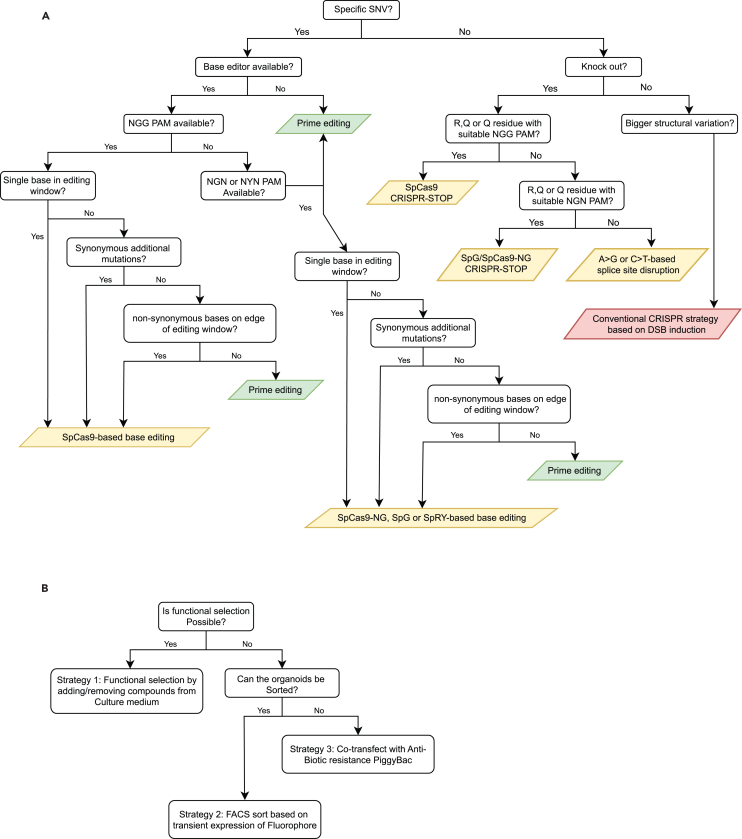
Decision tree to determine the optimal Next-Generation CRISPR tool for isogenic ASC line generation (A) Decision tree to choose the optimal tool to generate your mutation of interest. Although simplified, most mutations can be made by either base editing (yellow boxes) or prime editing (green boxes). The red box indicates conventional CRISPR with nuclease active Cas9. (B) Decision tree to choose the optimal selection strategy to select for CRISPR-edited organoids.

We recommend using canonical *Streptococcus pyogenes* Cas9 (SpCas9) fusion proteins for all next-generation CRISPR applications if an NGG protospacer-adjacent motif (PAM) is available. Only if non-canonical PAMs such as NGN or NYN are required we recommend using evolved SpCas9 variants such as SpCas9-NG or SpRY.^19^^,^^20^ Follow the flow diagram in Figure 1A to choose the right genome editing tool for your mutation of interest.

***Optional:*** In this protocol, we focus on the first developed base editors, being A>G and C>T and their application for disease modeling in ASC organoids. For a complete overview of available base editors we would like to refer to: Huang et al.^21^ and Rees and Liu.^22^Table 1 highlights base editors for most of the transition and transversion mutations.

**CRITICAL:**Table 1 highlights two kinds of C>T base editors. The base editor with restricted sequence context exhibits low editing efficiencies in a GC context. The evolved variants such as EvoFERNY and CBE6-variants do no longer have this sequence context. While we use AncBE4max for most experiments here, we recommend using either EvoFERNY or CBE6 to ensure base editing in all sequence contexts.

***Optional:*** In this protocol we describe the delivery of CRISPR tools via plasmids. Alternatively, recombinant protein purification protocols of base and prime editors have been described that can be combined with synthetic sgRNA’s that can be ordered at companies such as IDT and Synthego.^21^

**Table 1:** Base editors overview

Nucleotide changes	Base editors available
A>G	ABE7.10^13^ ABE8e^23^
C>T (Restricted sequence context)	BE3^12^ AncBE4max^24^
C>T (Unrestricted sequence context)	EvoFERNY^25^ CBE6^26^
C>G	CGBE^27^^,^^28^
A > Y (where Y is T or C)	AYBE^29^
G>T	gGBE^30^
T>G	DAF-BE^31^

### Strategy determination step 2: Picking the right ASC organoid selection strategy

**Timing: 30 min**

There are different methods for the selection of organoids after electroporation. In this protocol we exploit three different methods. We apply these methods in specific instances as highlighted in Figure 1B.

#### Functional selection

This is the most favorable option as it directly selects for organoids with your mutation of interest. It can be used when the edited gene gives growth independency to some medium components or small molecules. In the current study we apply this in the following two cases.

(1)WNT and Rspondin1 (Rspo1) are essential to sustain the growth of human colon organoids. When these organoids get edited for APC KO, the WNT pathway will be constantly active. As a consequence, there is no need to supply the medium with these two factors. Only the edited organoids will be able to survive and grow in the depleted medium (-WNT/Rspo1). (2)Similarly, organoids engineered with TP53 point mutation or KO can grow in the presence of Nutlin3a. In normal condition, the addition of Nutlin3a to the medium will cause the activation of TP53 leading to cell death. Mutations in other genes can also be selected for based on their effect on ASC organoid phenotype. The protocols we describe here can be used to select for the mutations in the Table 2.

**Table 2:** Functional selection for typical genes mutated in cancer

Mutated gene / pathway	Organoid type	How to select for this
KRAS (G12/G13 mutations)	Colon	Removal of EGF and addition of gefitinib^32^
BRAF	Colon	Removal of EGF and addition of gefitinib^33^
SMAD4	Colon	Removal of Noggin^32^ and A83-01^32^ from culture medium
TP53	All organoid types	Addition of Nutlin-3a to culture medium^32^
APC	Colon	Removal of Wnt and R-spondin from the culture medium^32^
PIK3CA	Colon	Removal of EGFR and addition of MEK inhibitors^34^

#### FACS-based selection

If a mutation cannot be selected for addition or removal of media components, organoids that have been transfected can be selected out of the bulk culture. Sorting of transfected organoids based on the transient GFP signal derived from the base/prime editor plasmid. Most of the CRISPR-based gene editors contain a GFP cassette in their sequence which will be expressed only when the cell is transfected. Thus, the presence of the GFP is a confirmation of the successful electroporation. Outgrowing organoid clones can then be clonally expanded and screened for your specific mutation of interest by Sanger sequencing. This strategy is, however, only applicable for organoid types that are resistant to the stress of FACS sorting. As FACS outgrowth efficiencies depend on organoid type and can differ between donors, this should be tested with the specific organoids that are used for genetic engineering.

#### Antibiotic selection

This form of selection is the least favorable option to select for transfected organoids and exploits is PiggyBac based random genomic integration of a resistance cassette to a specific antibiotic in the electroporated cells. In the current protocol we describe the use of a PiggyBac that renders the cells resistant to Hygromycin. The cells, with the integrated resistance, will survive to the addition of the antibiotic to the medium. Similarly to FACS-based selection of fluorophores, the clonally grown organoids can then be screened for your mutation of interest.

## Key resources table

**Table:** 

REAGENT or RESOURCE	SOURCE	IDENTIFIER
**Bacterial and virus strains**		

Subcloning Efficiency DH5α competent cells	Invitrogen	18265017
One Shot Mach1 T1 phage-resistant chemically competent *E. coli*	Invitrogen	C862003

**Biological samples**		

Human intestinal organoids	UMC	N/A
Human endometrium organoids	UMC	N/A
Human fetal liver organoids	LUMC	N/A

**Chemicals, peptides, and recombinant proteins**		

Advanced DMEM-F12	Thermo Fisher Scientific	12634-010
Penicillin-Streptomycin	Thermo Fisher Scientific	15140122
HEPES	Thermo Fisher Scientific	15630080
GlutaMAX	Thermo Fisher Scientific	35050061
B-27 supplement	Thermo Fisher Scientific	17504044
Nicotinamide	Sigma-Aldrich	N0636
N-acetyl-L-cysteine	Sigma-Aldrich	A9165
SB202190 (p38 inhibitor)	Sigma-Aldrich	S7076
EGF	PeproTech	AF-100-15
A83-01 (ALK4/5/7 inhibitor)	Tocris	2939
Prostaglandin E2	Tocris	2296
Y-27632 dihydrochloride (Rho-kinase inhibitor)	AbMole	M1817
Primocin	InvivoGen	ant-pm-2
FGF10	PeproTech	100-26
HGF	PeproTech	100-39H
FGF7	PeproTech	100-19
CHIR99021	Tocris	4423
Gastrin	Tocris	3006-1
TGF-α	PeproTech	100-16A
Wnt3A surrogate	U-Protein Express	Custom order
Noggin-Fc Fusion protein conditioned medium	U-Protein Express	Custom order
R-spondin1-conditioned medium	In-house production (see Pleguezuelos-Manzano et al.^35^)	N/A
TrypLE Express enzyme (1x), phenol red	Thermo Fisher Scientific	11568856
Dimethyl sulfoxide (DMSO)	Sigma	D8418
DAPI	Thermo Fisher Scientific	D1306
OptiMEM	Thermo Fisher Scientific	11058-021
Basement membrane extract (BME), type II	R&D Systems	3533-001-02
Hygromycin B Gold solution 100 mg/mL	InvivoGen	ant-hg-1
Nutlin-3 (10 mM stock)	Sigma	N6287
Q5 High-fidelity DNA polymerase	New England Biolabs	M0491L
T4 DNA ligase	New England Biolabs	M0202L
DpnI	New England Biolabs	R0176L
BbsI	New England Biolabs	R0539
T4 polynucleotide kinase	New England Biolabs	M0201L
TSAP	Promega	M9910
Bsa1-HFv2	New England Biolabs	R3733L

**Critical commercial assays**		

Quick Ligation Kit	New England Biolabs	M2200L
Quick/DNA Micro Prep	Zymo Research	D3021
NucleoSpin Gel and PCR Clean-up	Macherey-Nagel	740609.50
PureLink Quick Plasmid Miniprep Kit	Invitrogen	K210011
PureLink HiPure Plasmid Midiprep Kit	Invitrogen	K210004
PureLink HiPure Plasmid Maxiprep Kit	Invitrogen	K210006

**Oligonucleotides**		

Set of dATP, dCTP, dGTP, dTTP	Promega	U1420

**Recombinant DNA**		

pFYF1320	Fu Y et al.^36^	Addgene plasmid #47511
pSPgRNA	Perez-Pinera et al.^37^	Addgene plasmid #47108
pU6-tevopreq1-GG-acceptor	Nelson et al.^38^	Addgene plasmid #174038
pCMV_AncBE4max_P2A_GFP	Koblan et al.^24^	Addgene plasmid #112100
pCMV_SpCas9-NG_AncBE4max_P2A_ GFP	Geurts et al.^1^^,^^2^	
pCMV-PE6b	Doman et al.^39^	Plasmid #207852
Piggyback hygromycin	Geurts et al.^1^^,^^2^	
Piggyback transposase	Geurts et al.^1^^,^^2^	

**Software and algorithms**		

Benchling	N/A	https://www.benchling.com

**Other**		

FastDigest buffer 10x	Thermo Fisher Scientific	B64
EDTA TE buffer	G-Biosciences	786-150
10x Cutsmart buffer	New England Biolabs	B6004S
Nuclease free duplex buffer	IDT	11-01-03-01
48-well suspension culture plate	Greiner Bio-One	677102
12-well suspension culture plate	Greiner Bio-One	665102
15 mL conical tube	Greiner Bio-One	188271
50 mL conical tube	Greiner Bio-One	227261
Blue filter lid FACS tube (Falcon 352235)	Thermo Fisher Scientific	08-771-23
Gene Pulser/MicroPulser electroporation cuvettes, 0.2 cm gap	Bio-Rad	1652086
Cuvette Plus, 2 mm gap, 400 μL, Sterile Pkg/10, Blue	BTX	45-0135
NEPA21 super electroporator	Nepa Gene	N/A
Flow cytometry cell sorter (e.g., BD Influx")	BD Biosciences	N/A
Fluorescence and bright-field microscope (e.g., EVOS cell imaging system)	Thermo Fisher Scientific	N/A
NanoDrop	Thermo Fisher Scientific	N/A

## Materials and equipment

**Table:** ADF+++

Reagent	Final concentration	Amount
Advanced DMEM-F12	N/A	485 mL
Penicillin-Streptomycin 10,000 U/mL	100 U/mL	5 mL
HEPES 1 M	10 mM	5 mL
GlutaMax 200 mM	2 mM	5 mL

Media can be stored at 4°C for up to 1 month.

**Table:** Expansion medium Intestine (100 mL)

Reagent	Final concentration	Amount
ADF+++	N/A	75.4 mL
Wnt3A surrogate	0.15 nM	100 μL
R-spondin 1-conditioned medium	20% final volume	20 mL
Noggin-Fc Fusion Protein conditioned medium	1% final volume	1 mL
B-27 Supplement	2% final volume	2 mL
Nicotinamide 1 M	10 mM	1 mL
N-Acetyl-L-cysteine 500 mM	1.25 mM	250 μL
SB202190 (P38 inhibitor) 30 mM	3 mM	10 μL
EGF 500 μg/mL	50 ng/mL	10 μL
A83-01 (ALK4/5/7 inhibitor)5 mM	500 nM	10 μL
Prostaglandin E2 10 mM	1 mM	10 μL
Primocin 50 mg/mL	0.1 mg/mL	200 μL

Media can be stored at 4°C for up to 2 weeks.

**Table:** Expansion medium Endometrium (100 mL)

Reagent	Final concentration	Amount
ADF+++	N/A	75.31 mL
Wnt3A surrogate	0.15 nM	100 μL
R-spondin 1-conditioned medium	20% final volume	20 mL
Noggin-Fc Fusion Protein conditioned medium	1% final volume	1 mL
B-27 Supplement	2% final volume	2 mL
Nicotinamide 1 M	10 mM	1 mL
N-Acetyl-L-cysteine 500 mM	1.25 mM	250 μL
FGF10 100 μg/mL	100 ng/mL	100 μL
EGF 500 μg/mL	50 ng/mL	10 μL
A83-01 (ALK4/5/7 inhibitor)5 mM	1 μM	20 μL
Prostaglandin E2 10 mM	1 mM	10 μL
Primocin 50 mg/mL	0.1 mg/mL	200 μL

Media can be stored at 4°C for up to 2 weeks.

**Table:** Expansion medium liver (100 mL)

Reagent	Final concentration	Amount
ADF+++	N/A	82.04 mL
R-spondin 1-conditioned medium	15% final volume	15 mL
Noggin-Fc Fusion Protein conditioned medium	1% final volume	1 mL
B-27 Supplement	2% final volume	2 mL
Nicotinamide 1 M	2.5 mM	250 μL
N-Acetyl-L-cysteine 500 mM	1.25 mM	250 μL
FGF10 100 μg/mL	100 ng/mL	100 μL
EGF 500 μg/mL	50 ng/mL	10 μL
A83-01 (ALK4/5/7 inhibitor)5 mM	2 μM	20 μL
Gastrin	10 nM	10 μL
CHIR99021	3 μM	100 μL
FGF7 100 μg/mL	50 ng/mL	50 μL
HGF 100 μg/mL	50 ng/mL	50 μL
TGF-α 100 μg/mL	20 ng/mL	20 μL
Primocin 50 mg/mL	0.1 mg/mL	200 μL

Media can be stored at 4°C for up to 2 weeks.

**Table:** Fluorescence-activated cell sorting (FACS) buffer (10 mL)

Reagent	Final concentration	Amount
ADF+++	N/A	10 mL
Y-27632 dihydrochloride 10 mM	10 μM	10 μL
DAPI 2 mg/mL	0.2 μg/mL	10 μL

Prepare within 24 h before use and store at 4°C.

## Step-by-step method details

### sgRNA design for base editors for a specific nucleotide variant (SNV)

**Timing: 30 min**

The steps below outline the general rules of thumb for designing base editing sgRNA’s for introduction of a specific SNV.

Visualization of genomic sequences is essential in genome engineering projects. In our gene editing pipelines we employ the online sequence visualization tool **Benchling** for its capabilities in integrated gRNA design, annotation, and Sanger trace alignment. Thus, all subsequent steps of the protocol can be designed, documented and analyzed in the same on-line tool.

1. Visualize your target gene in benchling by clicking **New (+) > DNA Sequence > Import DNA Sequences.**
2. In the tab **Import from Database**, type your target gene and select the newest version of the reference genome. For our human organoids we use reference genome GRCh38, “*Homo sapiens”* and press **Search.**

***Note:*** After the search is completed, a new screen will open that highlights that your gene was found in Ensembl.

3. Import your sequence as “Genomic Sequence” with set nucleotide type to “DNA”.
  a. Press **Import.**

***Note:*** If the program does not return anything after pressing search during the import process, it may be that your gene of interest has an alternative name. Search in databases such as UniProt, Genecards or Ensembl for alternative names of your gene and try again.

4. Localize the SNV of interest in the gene of interest you imported into benchling.
5. Make an annotation of the point mutation.
  a. Click on the features tab in the top right of the screen.
  b. In the annotations tab, click on create new.
  c. Name the annotation appropriately.
6. Check whether there is an appropriate sgRNA available for base editing of your SNV of interest by following the steps below. These are also highlighted in Figure 1A.
  a. Check whether there is an NGG PAM available for your base edit of interest. Base editors function in an editing window that is dependent on the base editor your use. A general rule of thumb is that the editing window spans from position 4 to 8 in the spacer sequence Figure 2A.
    i. If no NGG PAM is available, check for availability of NGN PAMS. These can be targeted in combination with evolved Cas9 variants such as SpCas9-NG^20^ or SpG.^19^
    ii. If no NGN PAM is available, check for availability of NYN PAMs (where Y is A or C). These can be targeted with SpRY.^19^
    iii. If no PAM is available proceed with prime editing design as described later in this protocol.
      **CRITICAL:** Base editors only function on the PAM strand. It is possible to use a C>T base editor to do a G to A base edit by targeting the reverse strand. Examples of sgRNA’s on the forward and reverse strand can be found in Figures 2C, 2D, and 2E.
      **CRITICAL:** Base editors with restricted sequence context, such as AncBE4max, exhibit low editing efficiencies in a GC context. The evolved variants such as EvoFERNY and CBE6-variants do no longer have this sequence context. While we use AncBE4max for most experiments here, we recommend using either EvoFERNY or CBE6 to ensure base editing in all sequence contexts.

**Figure 2:**
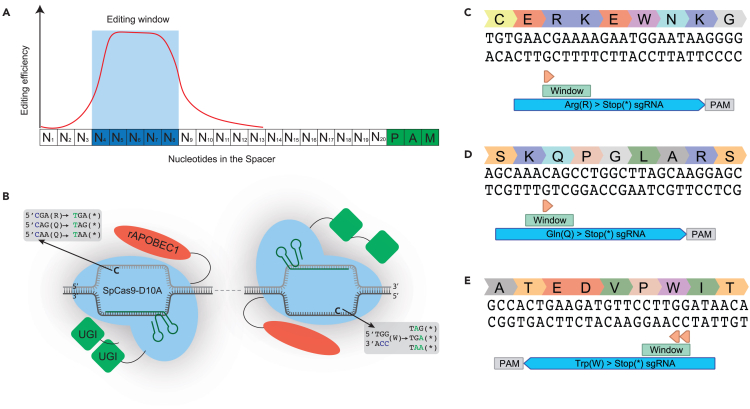
Base editing sgRNA design for specific SNVs and CRISPR-STOP (A) cytidine and adenine deaminases function only on single-stranded DNA. Therefore, base editor activity is limited to a small editing window within the R-loop that spans from roughly the 4th to the 8th base from the start of the sgRNA. (B–E) (B) Principles of CRISPR-STOP that allows for stop codon introduction on Arginine and Glutamine residues on the forward strand and on Tryptophan residues on the reverse strand. sgRNA design of CRISPR-stop in Benchling.com on (C) R (Argininine), (D) Q (Glutamine), and (E) Tryptophan (W) residues.
  b. Check whether there are additional bases in the editing window that can be targeted by your base editor.
    i. If there are additional bases in the window from position 4 to 8, check whether they result in a synonymous base change. If so, continue with the next steps.
    ii. If there are additional bases that cause missense or even nonsense mutations, there may not be a suitable sgRNA for base editing available for your target SNV. Proceed with prime editing design as described later in this protocol.
      ***Note:*** Sometimes there are additional bases in the editing window that cause an amino acid change. However, as indicated in Figure 2A, in most cases the editing window signifies a normal distribution of editing efficiencies indicating the highest editing efficiency on position 5 and 6 of the editing window. Thus, if the additional bases are on position 7 or 8 it may be worth it to proceed with base editing. In this protocol we will go through a clonal step and by screening a multitude of clones there is a high possibility of finding a clone in which base editing effectivity is lower and thus did not edit on the edges of the editing window.
7. Make an annotation of the 20 nucleotide sgRNA sequence in Benchling via the features tab on the top right. Make sure that the orientation, either forward or reverse strand is indicated correctly.
8. Proceed with cloning of your designed sgRNA according to the Inverse PCR protocol (steps 30 to 44) or the golden gate strategy (steps 45 to 53) depending on sgRNA availabilities in your lab.

### sgRNA design for CRISPR-STOP-mediated gene knock-out

**Timing: 30 min**

In the following section we describe how to design sgRNA to generate genes knock-out with Base editors. For this type of conversion we utilize C>T base editors.^40^ Prior to this, we recommend visualizing your gene of interest according to steps 1–3.

9. Select the optimal exon where your CRISPR-STOP sgRNA can be designed by adhering to the following set of rules.
  a. Do not put a sgRNA in the first coding exon of your target gene. The cell may use an alternative start site after your stop-codon. This would thus not result in knock-out of your target gene.
  b. Make sure the exon you choose is present in all transcripts of your target gene. Alternative splicing may result in different variants of your protein; putting it in an exon that is not present in all transcripts may not result in full gene knock-out. You can do this by visualizing your target gene in the Ensembl genome browser.^41^
    ***Optional:*** If possible, select an exon that has an incomplete codon (one or two remaining bases of a triplet) at either end of the exon-intron boundary. This will further diminish the chances that the cell can use alternative splicing to get rid of your induced stop codon.
10. Make sure that there are Glutamine (Q), Tryptophan (W) or Arginine (R) residues within your chosen exon.

***Note:*** C>T base editor mediated CRISPR-STOP can introduce stop codons on the complement/forward strand on Glutamine residues (CAA to TAA, CAG to TAG) and one Arginine residues (CGA to TGA). On the reverse strand Tryptophan residues (TGG to TGA, TAG or TAA) can be used (Figure 2B).

***Note:*** The following steps can also be performed without the use of the in-built CRISPR design tool of Benchling as long as you adhere to the base editing sgRNA design rules; editing window 4–8 nucleotides. Additional bases in the editing window are less important in this case as the end result will be a gene knock-out.

11. Select the exon you have chosen for your knock-out guide + 20 bases upstream and downstream. Click on the right side of the screen on the **target sign CRISPR** and click on **Design and Analyze guides.**
12. A new CRISPR design window will open.
  a. Tick the Guides for “base editing (Komor et al., 2016)” box under the design tab.
  b. Keep the Guide length at 20 nucleotides. Under the Genome tab select human (GRCh38).
  c. Click **Finish**.

***Note:*** Keep the PAM set to NGG (SpCas9, 3′ side) in the first round of sgRNA design. If no good sgRNA’s are designed in the process, you can change this to NG (SpCas9 NG, 3′ side) and perform the following steps again until a good sgRNA is designed.

***Note:*** The chance of nonsense mediated decay upon stop-codon induction is the highest if the codon is at least 50 base pairs upstream of an exon-exon junction.^42^ While we have seen efficient gene knock-out without taking this into account it may increase the chance of successful gene perturbation.

13. Click the blue **+** sign to **Create** and detect all PAM sequences and corresponding sgRNA sequences.
14. Scroll through the list on the right side of the window that contains all sgRNA sequences until you find a stop codon sign (∗) in a red box. This indicates that the selected sgRNA is compatible with CRISPR-STOP.
15. Based on the location within the editing window, for each C residue that may be edited a predicted editing efficiency is calculated *in silico.*

***Note:*** This is done based on the location within the editing window. Ensure that the C > T edit that results in the stop codon has a predicted efficiency of at least ∼10.

16. Click on the blue hyperlink value in the Off-Target column next to your sgRNA of interest and check which off-target loci the sgRNA could bind to.

***Note:*** Avoid choosing a sgRNA that binds to additional genes to increase specificity of your gene edit.

17. Create an annotation for your sgRNA in the created Benchling file.

***Note:*** Make sure to put the annotation on the reverse strand if your target CRISPR-STOP amino acid is a Tryptophan. Examples of designed CRISPR-STOP sgRNA’s for Arginine (Figure 2C), Glutamine (Figure 2D) and Tryptophan (Figure 2E).

**CRITICAL:** CRISPR-STOP sgRNA’s for Tryptophan residues are on the reverse complement strand. Make sure to copy this correctly. To reduce mistakes in copying, the annotations in the previous step serve as a reminder for sgRNA orientation and will reduce cloning mistakes.

18. The sgRNA spans the 20 nucleotides directly upstream of the PAM. Clone these 20 nucleotides according to either the “inverse PCR” protocol or the “Golden gate” protocol outlined below depending on sgRNA vector availability in your lab.
19. Proceed with cloning of your designed sgRNA according to the Inverse PCR protocol (steps 30 to 44) or the golden gate strategy (steps 45 to 53) depending on sgRNA availabilities in your lab.

### Genotyping primer design for validation of CRISPR edits

**Timing: 20 min**

The 3D matrix used for growing organoids is derived from mice and contains enough mouse DNA to be amplified by PCR. Consequently, it is crucial to design genotyping primers that specifically bind to human DNA and not to mouse DNA. Therefore, we recommend to design sequencing primers using a tool like Primer-BLAST (https://www.ncbi.nlm.nih.gov/tools/primer-blast/) as it can simultaneously BLAST both genomes.

20. Open a new tab in your browser (next to the benchling tab that has your sgRNA design) and go to Primer-BLAST.
21. Copy the 250 bases upstream- and 250 bases downstream of your designed sgRNA and paste this in the “**PCR Template**” box in Primer-BLAST.
22. In the “**Primer Parameters**” box set the “**PCR product size**” minimum to 400.

**CRITICAL:** To ensure specific PCR amplification over a wide range of targets we recommend to perform PCRs with a maximum length of 500, as specified by your PCR template of choice. Due to the short amplification time required, this drastically reduces unwanted aspecific amplification that may occur with longer amplification times.

**CRITICAL:** We do not recommend to perform Sanger sequencing on short PCR fragments. The required input DNA for fragments below 400 base pairs is so little that reproducibility goes down due to inaccurate DNA quantification or pipetting errors. Therefore, in this protocol we start the design at 400 base pairs and increase the size if no specific primers can be designed.

23. In the “Primer Pair Specificity Checking Parameters” box choose the option “Refseq representative genomes” under the “Database” tab.
24. Under the “**Exclusion Organism**” tab, “homo sapiens” is chosen by default. Click the “**Add organism**” button and fill out “Mus Musculus” in box that newly appears.
25. Scroll down and click the blue button “**Get Primers**”.
26. After a few seconds (depending on server use) a new screen will appear that indicates that your sequence is found in the human genome. Check in the “**Gene**” column on the right of the page if this is your target gene. If this is the case, tick the box on the left of the page to signify that this is your intended target and press the “**Submit**” button.

***Note:*** If multiple targets are found by BLAST it may be that your gene of interest has been duplicated in the genome. Check if the additional hits are pseudogenes or known protein coding genes. Depending on your research question this may require re-design of your sgRNA’s or your experiment.

27. A new window will appear that shows the designed primer pairs for PCR amplification of your target of interest. Select a primer pair and make annotations in benchling for the forward and reverse primer.

***Note:*** If primer-BLAST cannot design primers at your desired length, increase the amplicon size by copying a larger area around your designed sgRNA.

28. Pick a forward or reverse primer from a second primer pair designed by primer-BLAST as your sequencing primer.

**CRITICAL:** The first bases in a Sanger sequencing reaction are of low quality. To ensure high sequence quality at your target site, make sure that the Sanger sequencing primer is at least 80 base pairs up- or downstream of your sgRNA.

***Note:*** Since the sequencing primer is unlikely to bind to any unwanted non-specific products amplified during PCR, the chances of achieving high-quality sequencing are increased. Therefore, although not essential, we recommend using a sequencing primer to enhance the robustness of this protocol.

29. Purchase the forward, reverse and sequencing primer at your oligo distributor.

### Base editing gRNA plasmid generation: Inverse PCR protocol to clone base editing sgRNAs

**Timing: 4–5 days**

In this section we describe vector generation for sgRNA cloning using an inverse PCR reaction. The base vector for this cloning protocol is: pFYF1320 (Addgene #47511, a kind gift from Keith Joung). To clone subsequent sgRNA’s for base editing you can use any sgRNA as template that is based on pFYF1320. This protocol is based on the protocol described by Huang et al.^21^

**CRITICAL:** We recommend using Personal Protection Equipment (gloves and lab coat) and working in a sterile environment when handling cells or bacteria.

30. Order the following universal forward primer/oligo: "/5phos/ GTTTTAGAGCTAGAAATAGCAAGTTAAAATAAGGC. This is a universal primer that will remain constant for all sgRNA cloning reactions in the pFYF1320 backbone.
31. Order the reverse primer/oligo by pasting the reverse complement of your designed sgRNA in front of the following universal reverse primer part: CGGTGTTTCGTCCTTTCCACAAG.
32. Make the following PCR reaction master mix according to the manufacturer’s protocol. In this case we show the mix for Q5 (NEB). Other options would be Phusion (Thermo Fisher) or PrimeSTAR (Takara).

**Table:** PCR reaction master mix

Component	Volume
5X Q5 reaction buffer	10 μL
10 mM dNTPs	1 μL
10 μM Forward primer	2.5 μL
10 μM reverse primer	2.5 μL
10 ng Template DNA	2 μL
Q5 High-Fidelity DNA polymerase	0.5 μL
Nuclease free water	31.5 μL

**CRITICAL:** It is essential to use a high fidelity enzyme for this cloning Taq polymerases may introduce many mistakes during cloning and should thus be avoided.

33. Run the following PCR reaction on a thermocycler according to the PCR cycling conditions stated below.

**Table:** PCR cycling conditions

Step	Temp	Time
Denaturation	98°C	2 min
35 cycles	98°C	20 s
	60°C	30 s
	72°C	1:20 min
Final extension	72°C	2 min
Hold	4°C	∞

34. Run 5 μL of the PCR reaction on the 1% agarose gel for 45 min at 120 V and image the gel. One single band should now be observed at 2.2 kb.
35. Perform PCR cleanup using a PCR cleanup kit according to manufacturer’s recommendations. Elute the cleaned-up DNA in 20 μL.
36. Measure the concentration of the cleaned up PCR product using a nanodrop spectrophotometer.
37. Mix the following sgRNA ligation and template depletion reaction mix.

**Table:** sgRNA ligation and template depletion reaction mix

Component	Volume/Amount
10x T4 DNA ligase buffer (NEB)	1 μL
T4 DNA ligase (NEB)	1 μL
DpnI (NEB)	1 μL
100 ng Template (PCR cleanup)	X μL
Nuclease free water	7-x μL
Total	10 μL

**CRITICAL:** It is essential that both T4 Ligase and DpnI have 100% activity in the same buffer. We recommend using NEB as a supplier for those reactions.

38. Place the PCR strip in a thermocycler and run the following protocol to perform sgRNA ligation and template depletion.

**Table:** sgRNA ligation and template depletion cycling conditions

Temperature	Time	Description
22°C	15 min	15 min at room temperature (20°C–25°C) ensures T4 ligase activity and thus ligation of the construct
37°C	30 min	DpnI reaction time to get rid of initial PCR template material
80°C	20 min	Heat inactivation of DpnI, make sure this applies to your DpnI enzyme
4°C	∞	Hold

39. Transform 4 μL into 50 μL of your bacterial strain of choice (DH5α or Mach1) by using the following steps.
  a. Thaw the bacteria on ice.
  b. Add ligated plasmid and bacteria together on ice for 20 min.
  c. Heat shock the bacteria at 42°C for 45 s.
  d. Place back on ice for 2 min.
  e. Add LB without antibiotics and recover for 30–45 min (as these bacteria are Ampicillin resistant, this step is not needed, however, we notice increased efficiencies after recovery).
  f. Streak the bacteria on Ampicillin resistance plates and incubate overnight.

**CRITICAL:** The volume of DNA may never exceed 10% of the total volume of a transformation reaction. This is why we recommend to transform a maximum 4 μL into 50 μL of bacteria.

***Note:*** Alternatively 1 μL of ligated plasmid may be transformed into 10 μL of bacteria to reduce material costs.

40. To check for correct plasmids, the next day, pick 3 colonies, place them in 3 mL LB containing 100 μg/mL ampicillin and incubate overnight.
41. The next day, spin down 2 mL of the overnight culture and perform a miniprep to recover the bacteria for sequencing. Store the remaining 1 mL in the fridge for future inoculation for either midi or maxiprep.

**Pause point:** Bacteria in LB can be stored at 4°C for up to a month.

42. Analyze the contents of your Sanger sequencing with the following sequencing primer: 5′-GGGCAGGAAGAGGGCCTAT-3′.
43. Perform Sanger sequencing alignment in Benchling according to the steps below.
  a. Visualize your target gene in benchling by clicking **New (+) > DNA Sequence > Import DNA Sequences.**
  b. In the tab **Import from Database**, copy the following Addgene plasmid code: https://www.addgene.org/47511/ and click import.
  c. Replace the 20 nucleotides directly upstream of the sgRNA backbone with your guide RNA sequence.
  d. Click on **Alignments > Create new alignment** on the right side of the screen.
  e. Drag and drop your Sanger sequencing .ab1 files into the screen and click **Next.**
  f. In the new screen that opens click **Create alignment.**
  g. Check in the alignment if the 20 nucleotides of the sgRNA are now present in the sgRNA vector.
44. After sequencing has shown that the correct sgRNA plasmid is generated, perform a Midiprep according to manufacturer’s recommendation to recover a sufficient amount of plasmid DNA for subsequent transfections.

**CRITICAL:** Make sure the concentration of the sgRNA vector after midiprep is at least 1 μg/μL to reduce volume of liquid used in future steps.

### Base editing gRNA plasmid generation: Golden gate BsbI protocol to clone base editing sgRNAs

**Timing: 4–5 days**

As an alternative to the inverse PCR cloning strategy described above, here, we describe the generation of sgRNA vectors using golden gate BsbI cloning. The base vector for this cloning protocol is: pSPgRNA plasmid (Addgene #47108). This protocol is based on Perez-Pinera et al.^37^

45. To order oligos for golden gate cloning take the complement and reverse complement strands of your sgRNA and add the following overhangs.

**Table:** Primer sequences

name	Sequence
Forward guide	5′ – CACCGNNNNNNNNNNNNNNNNNNN – 3′
Reverse guide	3′ – NNNNNNNNNNNNNNNNNNNCAAA – 5′

46. Linearize the pSPgRNA plasmid by preparing the following restriction reaction.

**Table:** pSPgRNA linearization restriction reaction mix

Component	Volume
1 μg of pSPgRNA plasmid	X
BbsI enzyme (NEB)	1 μL
TSAP (Promega)	1 μL
FastDigest Buffer 10X (Thermo Scientific)	2 μL
Nuclease free water	16 - X μL
Total volume	20 μL

47. Linearize the pSPgRNA plasmid by according to the pSPgRNA linearization thermocycling conditions.

**Table:** pSPgRNA linearization Thermocycling conditions

Temperature	Time
37°C	30 min
74°C	20 min

48. Purify the linearized backbone, for example by using the QIAquickPCR purification kit (QIAGEN 28104) according to manufacturer’s instructions. Elute the DNA using 30 μL low EDTA TE buffer (G-Biosciences 786–150). Measure the DNA concentration using a spectrophotometer (e.g., nanodrop).
49. Reconstitute the guide oligonucleotides to a concentration of 100 μM using low EDTA TE buffer.
50. Phosphorylate and anneal the forward and reverse guide oligonucleotides by making the pSPgRNA oligo annealing mix and running it according to the pSpgRNA oligo annealing thermocycler conditions.

**Table:** pSPgRNA oligo annealing mix

Component	Volume
T4 DNA ligase buffer 10X (NEB)	1 μL
T4 polynucleotide Kinase (NEB)	0.5 μL
Forward oligo 100 μM	1 μL
Reverse oligo 100 μM	1 μL
Nuclease free water	6.5 μL
Total volume	10 μL

**Table:** pSPgRNA oligo annealing thermocycling conditions

Temperature	Time
37°C	30 min
95°C	5 min
25°C	Ramp down from 95 degrees at 0.1°C per second.
22°C	∞

51. Pipette the pSPgRNA plasmid ligation mix and keep it at room temperature (20°C–25°C) for 10 min.

**Table:** pSPgRNA plasmid ligation mix

Component	Volume
50 ng linearized plasmid from **step 38**	X
Oligonucleotide duplex from **step 40**	1 μL
Quick ligation buffer (NEB M2200)	5 μL
Quick ligase (NEB M2200)	1 μL
Nuclease free water	4 – X μL
Total volume	11 μL

52. Transform 3 μL of this reaction mixture into bacteria of choice according to **step 39.**
53. Verify correct sequence clones followed by midiprep of a correct bacterial colony according to **40–44.**

### Prime editing guide RNA design

**Timing: 30 min**

**Figure 3:**
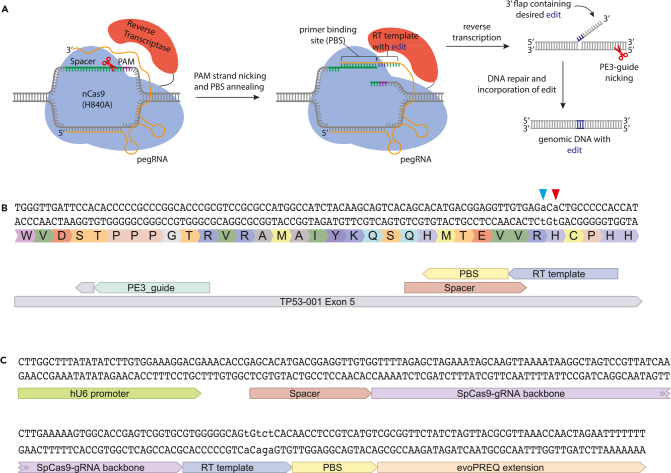
Prime editing sgRNA design (A) Principles of prime editing. The nickase SpCas9(H840A) nicks the PAM strand upon which the PBS in the 3′ extension of the pegRNA stabilizes the single stranded DNA break. The reverse transcriptase, attached to the Cas9 then uses the RT template of the 3′ extension to directly write your edit of interest into the target site. To further enhance editing efficiencies a second PE3 sgRNA nicks the unedited strand, which guides the cell to use the just edited strand as template for repair. (B) Screen capture of Benchling.com showing the orientation and design of a combination of pegRNA and PE3 guide that is used in this study to induce the TP53 R175H mutation in hepatocyte organoids. Red arrow indicates the target base change; blue arrow indicates a PAM disruption mutation that can sometimes be made to disable prime editor binding at the target site upon successful gene editing. (C) Screen capture from Benchling.com showing the orientation of Spacer, PBS and RT template in the pegRNA.

Prime editors have significantly increased the target scope of DSBs-free genome engineering and can be used in cases where no suitable base editing sgRNA can be designed or if the target mutation is an indel, small insertion or deletion. Prime editors consist of a nickase Cas9 (Cas9(H840A)) fused to a reverse transcriptase. The reverse transcriptase, that can turn RNA into DNA, finds its template in an elongated sgRNA called the prime editing guide RNA (pegRNA). The 3′ end of the pegRNA consists of a PBS sequence and an RT sequence. The PBS sequence binds and stabilizes the single strand of DNA that is overhanging after Cas9(H840A) has cleaved the target site. The RT then directly writes your edit of interest by using the RT template of the 3′ extension. Lastly, in the development of prime editing the same principles as in base editing are applied; a second guide RNA (called the PE3 or PE3b guide) cleaves the opposite strand, guiding the cell into using the newly edited strand as template for repair^14^ (Figure 3A).

**CRITICAL:** While prime editing is a very versatile technique, there are many variables that all influence the editing efficiency of the technique. The length of the PBS, the length of the RT and the distance between nicks of the pegRNA and PE3 guide all play a role. Thus, we recommend to first test for available sgRNA’s for base editing before opting for prime editing.

54. Localize the SNV or indel of interest in the gene of interest you imported into benchling.
55. Make an annotation of the point mutation.
  a. Click on the **features** tab in the top right of the screen.
  b. In the annotations, tab click on **create new.**
  c. Name the annotation appropriately.

**CRITICAL:** Because of the many variables, we highly recommend using online tools that have been trained by machine learning to design your prime editing guide RNA’s. Tools such as OPED^43^ or PRIDICT^44^ have been trained on vast *in vitro* datasets. Alternatively, tools that do not use machine learning, such as PegFinder^45^ or PrimeDesign^46^ can be used. These tools just adhere to the general rules of prime editors as described in: Anzalone et al.^14^

56. Use one of the tools described above to design your pegRNA and PE3 guide according to the rules below (Figures 3B and 3C).
  a. Prime editors can edit directly downstream of the single stranded DNA cut that is induced by the Aspartate residue on position 10 of the Cas9. This is always between the 3^rd^ and 4^th^ base upstream of the PAM. Editing efficiencies are highest if your target base is directly, as close as possible, to this cut.
  b. Extend the RT-part of the pegRNA 10 nucleotides beyond your edit of interest. As this has been found to be optimal for most point mutations and indels. If the goal is to insert or delete larger portions of DNA (from 10 base pairs onwards) include an extended RT of at least 25 nucleotides^47^
    **CRITICAL:** the first base directly downstream of the scaffold in the pegRNA may never be a C as this can interfere with the 3D structure of the sgRNA backbone and decrease editing efficiencies.^14^ As the RT is directly attached to the scaffold, make sure that it does not start with a C. If an RT extension of 10 beyond your target edit results in a C, extend it one further, to 8 bases.
  c. As a rule of thumb, start using PBS lengths around 13 base pairs as PBS lengths for most pegRNA’s are around this length.
  d. Choose a second sgRNA, called the PE3 guide, that is on the opposing strand. Choose a PE3 guide that cleaves between 50 and 100 nucleotides up- or downstream of the nick of the pegRNA.
    ***Optional:*** Sometimes there is an option to design the PE3 guide directly opposite of the pegRNA. This is called a PE3b guide and will only cleave the opposing strand once the prime editor has installed its target mutation. Whenever it is possible to use a PE3b guide do so.
    ***Note:*** We recommend always designing at least 2 combinations of PegRNA/PE3-guide to increase the chances that one of the two will work. It has been described that it would even be better to screen a plethora of guide combinations to find the most optimal combination for your target of interest.^18^ It depends on your research question whether this is worth the effort.
57. Make an annotation of the 20 nucleotides spacer sequence in benchling via the **features** tab on the top right. Make sure the orientation, either forward or reverse strand is indicated correctly.

**CRITICAL:** As prime editors can edit on the last 3 positions of your guide RNA, you need to make sure that the oligos you order for the spacer sequence do not contain your edit of interest. Mismatches in the sgRNA in the seed sequence directly upstream of the PAM will result in the inability of Cas9 binding to its target site.

58. Make an annotation of the 3′ extension of the pegRNA that contains both the RT and PBS.

**CRITICAL:** Due to the nature of the prime editor, the 3′ extension is always on the opposing strand in your pegRNA. The order in your final construct should always be: Spacer-Scaffold-RT-PBS. This can only be achieved by copying the sequence on the opposite strand compared to the spacer sequence.

59. Make an annotation of the PE3 or PE3b guide RNA.

### Golden gate cloning protocol for prime editing sgRNAs

**Timing: 4–5 days**

The PE3 sgRNA can be cloned according to either of the two protocols described above. The PegRNA cloning protocol is outlined below. We recommend cloning PegRNA sequences in evolved PegRNA backbone pU6-tevopreq1-GG-acceptor (Addgene #174038) as this will include PegRNA extensions that further stabilize the guide which results in higher editing efficiencies.^48^

This protocol is adapted from: Anzalone AV et al., Nature (2019).^14^

60. Order complement and reverse complement sequences of both the spacer as well as the extension with the standardized pegRNA overhangs indicated in the table below.

**Table:** Standardized pegRNA overhangs

name	5′	3′
top_spacer	caccg	gtttt
Bottom_spacer	ctctaaaac	c
Top_extension	gtgc	
Bottom_extension	cgcg

61. Purchase the universal SpCas9 sgRNA backbone/scaffold sequences with 5′ phosphorylated ends as indicated below.

**Table:** Primer sequences

name	Sequence 5′-3′
Scaffold_top	/5phos/AGAGCTAGAAATAGCAAGTTAAAATAAGGCTA GTCCGTTATCAACTTGAAAAAGTGGCACCGAGTCG
Scaffold_bottom	/5phos/GCACCGACTCGGTGCCACTTTTTCAAGTTGA TAACGGACTAGCCTTATTTTAACTTGCTATTTCTAG

62. Perform a restriction digest on the GG-acceptor according to the pegRNA-GG-Acceptor digestion master mix.

**Table:** pegRNA-GG-Acceptor digestion master mix

Component	Volume
6000 ng pU6-tevopreq1-GG-acceptor	X μL
Bsa1-HFv2 (NEB)	3 μL
10x Cutsmart Buffer	9 μL
Nuclease free water	78 - X μL
Total volume	90 μL

63. Run the reaction mixture on a 1% agarose gel and perform gel extraction on the 2.2 kb band according to manufacturer’s protocol.
64. Dilute the 2.2 kb piece to 30 ng/μL and store at −20°C for up to 6 months.

***Note:*** In the above steps you make enough linearized pegRNA vector backbone for hundreds of reactions, aliquot well and avoid freeze thawing to keep the restriction overhangs intact.

65. Mix the top and bottom oligos for the spacer, scaffold and backbone according to the Oligo Annealing reaction mix below.

**Table:** Oligo Annealing reaction mix

Component	Volume
Top oligo 100 μM	1 μL
Bottom oligo 100 μM	1 μL
Nuclease free duplex buffer	23 μL
Total volume	25 μL

66. Anneal the oligos by heating in a thermocycler to 95°C and slowly (0.1 degree per second) ramping down to 22°C.
67. Add 75 μL of Nuclease free water to the annealed oligos. They will now have a final concentration of 1 μM which is perfect for the final golden gate cloning reaction.
68. Assemble the PegRNA golden gate reaction mix for each pegRNA in a PCR strip as indicated below.

**Table:** PegRNA golden gate reaction mix

Component	Volume
Linearized pU6-tevopreq-1-GG-acceptor (30 ng/μL)	1 μL
Annealed spacer	1 μL
Annealed scaffold	1 μL
Annealed pegRNA extension	1 μL
BsaI-HFv2 (NEB)	0.25 μL
T4 DNA ligase (NEB)	0.50 μL
10x T4 DNA ligase buffer (NEB)	1 μL
Nuclease free water	4.25 μL
Total volume	10 μL

69. Perform a golden gate reaction in a thermocycler by running the pegRNA golden gate reaction program indicated below.

**Table:** pegRNA golden gate reaction

Temperature	Time
22°C	15 min
37°C	15 min
80°C	15 min
4°C	∞

70. Transform 4 μL of this reaction mixture into 50 μL bacteria of choice according to **step 39**.
71. Verify correct sequence clones followed by midiprep of a correct bacterial colony according to **steps 40–44**.

### Transfection of CRISPR tools into organoids by electroporation

**Timing: 1.5–2 h**

In this section we describe delivery of Next-generation CRISPR tools into organoids by electroporation using the NEPA21 electroporator. If this machine is not available for use, alternative strategies have been described such as Lipofectamine 2000 spinoculation,^32^ the Neon electroporator,^49^ or Lonza Nucleofection.^50^ We recommend to keep the preparation of organoids the same and only change electroporation conditions according to manufacturer’s instructions. In this protocol we describe the transfection of human colon, endometrium and hepatocyte organoids. The protocol is also adaptable to organoids derived from other organs. Additionally, it is adaptable depending on your selected strategy to select for CRISPR-edited clones, either based on phenotypic function of your mutation of interest or transfection.

**CRITICAL:** a) Passage organoids 5-7 days prior to electroporation as per usual. b) Use at least 200 µL (maximum 400 µL) of 3D-matrix containing a high density of organoids per electroporation condition. This corresponds to roughly 1 million cells per condition. if you are unsure about the amount of cells, count the cell prior to electroporation. c) Every buffer used during electroporation should be kept on ice and should be supplemented with 10µM Y-27632 to reduce cell death during the procedure.

72. **24 h before electroporation:** Supply the organoid expansion medium with 10 μM Y-27632 and 1.25% (vol/vol) DMSO.
73. **1 h before electroporation:** Pre-warm TrypLE solution supplemented with 10 μM Y-27632 to 37°C. Set the centrifuge to 4°C.
74. Prepare the plasmid DNA mix for the electroporation in a 1.5 mL tube as follows.
  a. For Base editors experiments.

**Table:** 

Plasmids	PiggyBac selection	Functional / FACS-based selection	Negative control
Cas9-Base editor	7.5 μg	10 μg	x
sgRNA	2.5 μg	3.5 μg	2.5 μg
Piggybac transposase	5 μg	x	x
Piggybac Hygromycin	5 μg	x	x

    ***Note:*** to multiplex sgRNA’s, add 2.5 μg or 3.5 μg of sgRNA plasmids on top of the Piggybac or Functional mix respectively.
  b. For Prime editors experiments.

**Table:** 

Plasmids	PiggyBac selection	Functional/ FACS-based selection	Negative control
Cas9-Prime editor	7.5 μg	7.5 μg	7.5 μg
PEgRNA	2.5 μg	2.5 μg	x
PE3	1.5 μg	1.5 μg	x
Piggybac transposase	5 μg	x	x
Piggybac Hygromycin	5 μg	x	x

    **CRITICAL:** We have not seen a significant decrease in survival rate of organoids in electroporations with up to 25 μg of CRISPR vectors. If you desire to multiplex more sgRNA’s, use less of each sgRNA to stay below 25 μg of total DNA.
    **CRITICAL:** Always include a negative control to assess the efficiency of the selection by using only the base editor or the sgRNA.
75. Look at your organoids prior to transfection under a brightfield microscope. Organoids should look healthy and undifferentiated Figure 4A.

**Figure 4:**
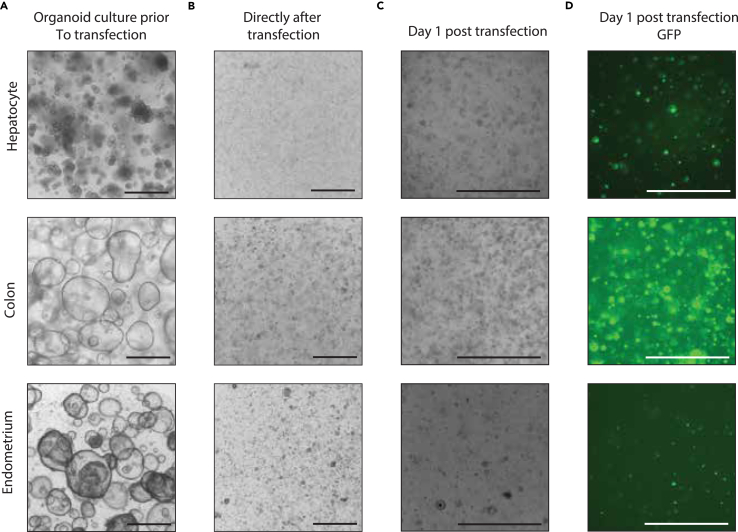
Delivery of plasmids encoding next-generation CRISPR tools in various ASC organoids (A) Bright-field image of hepatocyte, colon and endometrium organoids right before transfection by electroporation. (B) Bright-field image of hepatocyte, colon and endometrium organoids right after transfection by electroporation. (C) Bright-field image of hepatocyte, colon and endometrium organoids 24 h after transfection. (D) Fluorescent microscope image of GFP positive cells that indicate transfection. Scale bar = 1 mm.
76. Remove medium from the organoid wells, corresponding to 200 to 400 µL and add ADF+++ supplemented with 10 μM Y-27632 to collect the organoids. Pipette up and down with a P1000 tip to disrupt the 3D-matrix.

***Optional:*** Coat the pipet tip with FCS to prevent the organoids from sticking in the tip.

77. Spin down organoids at 500 × g for 5 min at 4°C.
78. Remove supernatant and add 2 mL of prewarmed TrypLE and resuspend organoids by pipetting using a P1000 tip.
79. Incubate the suspension at 37°C in a water bath for 5 min.

***Note:*** The time of dissociation can vary depending on the type of organoid. We advise to keep organoids in TrypLE not longer than 15 min to prevent cell death.

80. Resuspend organoids by using a P1000 tip and check the organoid size under a microscope. Aim for organoid fragments between 10 to 15 cells to achieve a high survival rate during electroporation.
81. When the desired cell clusters of 10 to 15 cells are acquired, continue with the protocol. If the disrupted organoid fragments are still too big, repeat steps 69–70 one or two more times (for max 15 min incubation time).
82. To inactivate TrypLE, add 10 mL of ice-cold ADF+++ supplemented with 10 μM Y-27632 to the cell suspension.
83. Spin down the cells at 500 × g for 5 min at 4°C.
84. Remove the supernatant and wash the pellet in 4 mL of OptiMEM supplemented with 10 μM Y-27632.
85. Spin down the suspension at 500 × g for 5 min at 4°C.
86. After the centrifuge step, resuspend your pellet in 100 μL of Opti-MEM supplemented with 10 μM Y-27632 per 200 to 400 μL of organoid starting material and keep the cells on ice.
87. Mix the cell suspension with the plasmid mix prepared in step 74.
88. Transfer the mix into an electroporation cuvette suitable for mammalian cells (2 mm distance between electrodes).
89. Electroporate using the following parameters, depending on your organoid type.

**Table:** Electroporation parameters

	Poring pulse colon	Poring pulse endometrium	Poring pulse hepatocyte	Transfer pulse (all types)
Voltage	175 V	150 V	175 V	20 V
Pulse length	5 ms	5 ms	7.5 ms	50 ms
Pulse interval	50 ms	50 ms	50 ms	50 ms
Number of pulses	2	2	2	5
Decay rate	10%	10%	10%	40%
Polarity	+	+	+	+/−

***Note:*** The parameters voltage and pulse length of the poring pulse can vary based on the type of organoids this should be optimized for the specific organoid culture in use.

***Note:*** Right before electroporation, we recommend measuring the impedance (Ω) on the machine. This value will give an idea of the quantity of DNA present in the cuvette. The best range for this value is between 0.035 and 0.055.

90. Immediately after the electroporation, add 400 μL of room temperature Opti-MEM supplemented with 10 μM Y-27632 to the cuvette.
91. Incubate the electroporated cells in the cuvettes for 30 min at room temperature (20°C–25°C). In this time the cells can recover from the electroporation which results in increased organoid outgrowth.
92. Pre-coat the sterile pipette that is supplied with the electroporation cuvette in Opti-MEM.
93. Using the pre-coated sterile pipette, resuspend the cells in the cuvette and transfer it to a 1.5 mL Eppendorf tube.
94. Spin down the electroporated cells at 500 × g for 5 min at 4°C.
95. Plate the organoids in 3-D matrix droplets very densely. Density changes between organoid lines and has to be tested for the line of choice (Figure 4B). For the organoids in this protocol, we suggest plating a single electroporation reaction (from 200 μL of starting material) in 240–320 μL of 3-D matrix.
96. Solidify the 3-D matrix by incubating your plate for 15–30 min at 37°C.
97. Add Expansion medium supplemented with 10 μM Y-27632.
98. 24 h after the electroporation, check for the presence of GFP signal using a microscope. When present, this will confirm that the electroporated plasmids are present in the cells Figures 4C and 4D.

***Note:*** Most of the base/prime editors plasmids contain a GFP cassette which gets expressed only when the plasmid is delivered in the cells.

### Functional and antibiotic selection CRISPR-edited organoids

**Timing: 1–1.5 h**

For functional selection we use human colon organoids electroporated with base editors to generate the mutation APC Q1046∗ and human fetal liver organoids engineered for TP53 R175H with prime editor. Antibiotic selection was performed in human endometrial organoids engineered for PTEN Q245∗ using base editor. We recommend performing the following single cell step 4–5 days after the electroporation.

**CRITICAL:** For any functional selection strategy it is essential to perform the steps below on control organoids at the same time. Without a proper control you are not able be sure that your experimental conditions survive selection, resulting in significant numbers of false positive organoids.

99. 4–5 days after electroporation, remove medium and add cold ADF+++ to the electroporated and non-electroporated (control) organoids.
100. Pipette up and down with a P1000 to disrupt the 3D-matrix.
101. Collect the organoids in a falcon tube and spin down at 500 × g for 5 min at 4°C.
102. Aspirate the supernatant and resuspend the cell pellet in 1 mL of prewarmed (37°C) TrypLE.
103. Incubate for 5 min at 37°C. Check the dissociation status under the microscope. In the presence of big clumps after 5 min, resuspend your cell suspension well and repeat step 103 again (we suggest to digest the samples not more than 15 min).

**CRITICAL:** Make sure to create a single cell solution during this step to ensure a clonal culture in the end.

**Figure 5:**
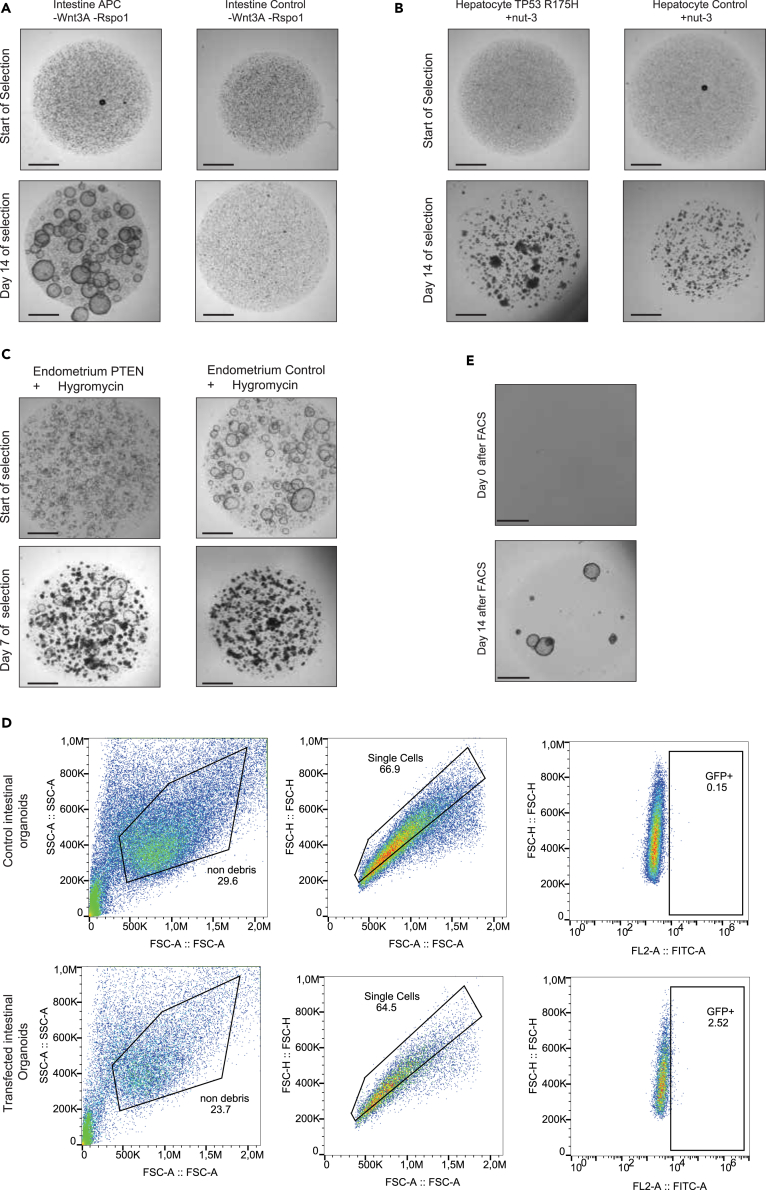
Selecting mutated ASC organoids based on function or transfection (A) Functional selection of APC mutants in colon organoids by withdrawal of Wnt3A and Rspo1 from the culture medium. (B) Functional selection of TP53 mutant hepatocyte organoids by addition of nutlin-3 to the culture medium. (C) Transfection selection of PTEN mutant endometrium organoids by addition of hygromycin to the culture medium. (D) FACS Strategy to select of transfected, GFP+ve adult stem cells. (E) Colon organoid outgrowth after FACS sorting. Top image shows the organoids right after plating, bottom image shows the clonal outgrowth 14 days after FACS. Scale bar = 2 mm.
  b. ***For antibiotic selection***: add the desired antibiotic to the expansion medium (for this protocol we used hygromycin 1:1000) (Figure 5C).
111. Add the selection medium mixed for the desired selection to the solidified 3D matrix containing the single-cell suspension.
112. Refresh the medium every 3 days until the selection is completed. The selection is done when the control organoids are completely dead while clonal organoids are visible in your experiment (Figures 5A–5C). Proceed with step 133 when this is achieved.

***Note:*** Once the control organoids are completely dead, it may take a while for your clones of interest to grow sufficiently for propagation. In these cases we advise to add full expansion medium to the selection plates to increase the growth speed of the organoids.

***Note:*** Usually it takes about 10–14 days to see the effects of functional selection. Antibiotic selection (in our case, hygromycin), is generally quicker and takes ∼8 days. Sometimes functional selections can take more time. In those cases, organoids have to be splitted (repeat the protocol above) before proceeding to the next step. Perform this split if the control organoids are continuing to grow after 14 days.

104. To inactivate TrypLE, add 10 mL of ice-cold ADF+++ to the cell suspension.
105. Centrifuge at 500 × g for 5 min at 4°C.
106. Aspirate the supernatant carefully and leave 20–30 μL of liquid on top of your pellet.
107. Resuspend the organoids in the 3D-matrix. Adjust the gel’s volume needed for plating accordingly to the cell pellet.
108. Plate the organoids in a 24-well or 12-well plate.
109. Let the 3D-matrix solidify for 15–30 min at 37°C in the incubator.
110. In the meantime, prewarm the selection medium in a water bath at 37°C.
  a. ***For functional selection***: prepare expansion medium depleted of specific growth factors or supplied with small molecules according to Table 2 (Figures 5A and 5B).

### Fluorescence-activated cell sorting (FACS) selection

**Timing: 2 h**

For this part we show the FACS selection of human colon organoids electroporated for APC Q1046∗ base editor. This enables us, contrary to the functional selection described above, to generate heterozygous APC organoid lines. Some organoid types, such as the hepatocyte cultures described in this protocol, cannot be FACS sorted. Make sure to test viability after FACS sorting for your organoid line of choice prior to doing this protocol.

113. Three to four days post electroporation GFP positive cells can be sorted by FACS.

**CRITICAL:** In our experience FACS sorting after 1 or 2 days does not result in CRISPR-edited organoids. While the percentage of green cells may be higher at day 2, CRISPR-editing is incomplete and subsequent Sanger sequencing will result in wild-type clones.

114. Remove medium and add cold ADF+++ to the electroporated organoids. Also do this for non-electroporated organoids to use as a negative, non fluorescent, FACS control.
115. Pipette up and down with a P1000 until the 3D-matrix is disrupted.
116. Collect the organoids in a falcon tube and spin down at 500 × g for 5 min at 4°C.
117. Aspirate the supernatant and resuspend the cell pellet in 1 mL of prewarmed (37°C) TrypLE.
118. Incubate for 5 min at 37°C. Check the dissociation status under the microscope. In the presence of big clumps after 5 min, resuspend your cell suspension well and repeat step 118 again (we suggest to digest the samples not more than 15 min).

***Note:*** To FACS sort we want to achieve a single cell suspension. Big clumps are not suitable for this type of experiment.

119. To inactivate TrypLE, add 10 mL of ice-cold ADF+++ to the cell suspension.
120. Centrifuge at 500 × g for 5 min at 4°C.
121. Remove the supernatant and resuspend the cell pellet in 400 μL cold ADF+++.
122. Transfer the cells in a FACS tube with a blue filter (Falcon #352235) to filter out the leftover cell-clumps.
123. For the collection of the sorted cells: prepare 1.5 mL Eppendorf tubes containing 500 μL ADF+++ supplemented with 10 μM Y-27632.

***Optional:*** Incubate your cell suspension with DAPI for live dead cell gating strategy.

124. Sort single living cells based on GFP expression. Use the negative control to design correct sorting gates (Figure 5D).

***Note:*** Outgrowth efficiency varies between different organoid lines. The optimal cell density for plating is 10 cells/μL. Thus, we advise to sort at least 1000 cells (best range is between 1000–3000 cells). The lowest amount to sort for organoid formation is 500 cells.

125. Centrifuge the collected cells at 500 × g for 5 min at 4°C.
126. Aspirate the supernatant carefully and leave 20–30 μL of liquid on top of your pellet.

**CRITICAL:** The cell pellet is not visible at this point. So, it is crucial to remove the supernatant slowly and carefully to avoid the loss of the cells.

127. Resuspend the cells in 3D-matrix. Adjust the gel’s volume accordingly to the amount of cells sorted (cell density 10 cells/ μL. E.g. if sorted 2000 cells, 200 μL of 3D-matrix is needed for plating).
128. Plate the cells in 3D-matrix into a 24-well or 12-well plate (Figure 5E).
129. Let the 3D-matrix solidify for 15–30 min at 37°C in an incubator.
130. In the meantime, prewarm expansion medium supplemented with 10 μM Y-27632°C to 37°C.
131. Add expansion medium to the well.
132. Refresh wells every 3 days with expansion medium supplemented with 10 μM Y-27632 until the organoids are grown for the generation of a clonal line (Figure 5E). After organoids have grown, proceed with step 133 for clonal expansion and genotyping.

### Clonal expansion of CRISPR-engineered organoid lines

**Timing: 1–1.5 h**

In this chapter, we show the final step of the protocol: how to generate clonal lines after the selection step and how to determine whether the gene of interest has been successfully edited. By following these steps it is possible to genotype your clones within 2 days after organoid picking and expansion. To ensure smooth transition into genotyping, make sure to follow steps 20–29 that highlight genotyping primer design.

**Figure 6:**
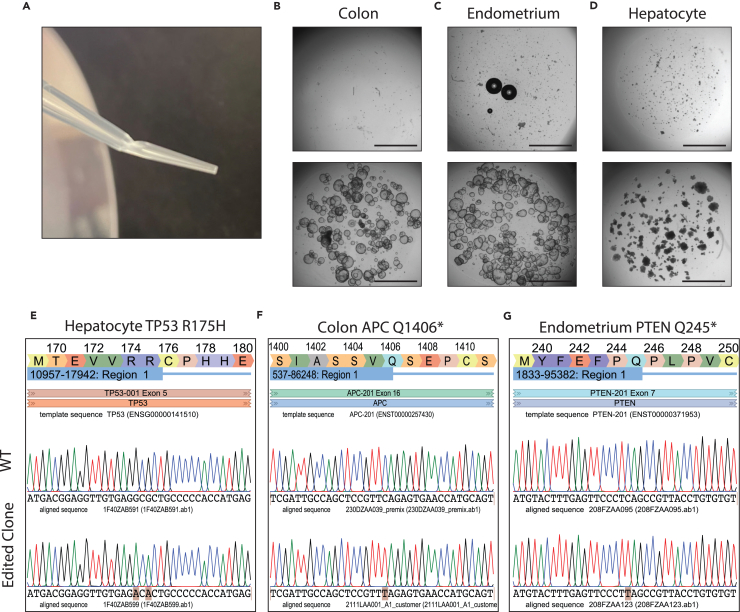
Clonal passaging of engineered organoids and sanger sequence validation (A) Bent pipette tip that is optimal for picking single organoid clones after any type of selection. This allows for an easy reach into the well. Bright-field images of Colon (B), Endometrium (C) and Hepatocyte (D) organoids right after picking and after outgrowth. Scale bar = 2000 μm. Sanger sequencing trace of prime edited hepatocyte organoids (E) and base edited colon (F) and endometrium (G) organoids and their respective wild-type counterparts. Direct screen captures from benchling.com.
135. Working under the microscope, use the bent pipette tip to pick an individual organoid from the plate and transfer it to a 1.5 mL Eppendorf tube.

133. Prewarm in TrypLE supplemented with 10 μM Y-27632°C to 37°C.
134. Bend a p200 pipette tip to a 45° angle (Figure 6A).

**CRITICAL:** It is important to pick a single organoid to generate a clonal line.

This step is also contamination prone, since we are working in an open environment not under the sterility of a hood. Therefore, we suggest opening and closing the lid of the plate quickly to reduce the exposure to air.

136. Repeat the previous step for 12 to 24 clones.

***Note:*** The optimal number of picked organoids will depend on the efficiency of the sgRNA and the outgrowth efficiency after picking.

137. Add 100 μL of prewarmed TrypLE to each picked clone.
138. Incubate the samples in the water bath at 37°C for 5 min.
139. Dissociate the organoid properly by pipetting the solution up and down 20 times using a p200 followed by vortexing for 10–20 s.
140. Check the dissociation process under the microscope. If the organoids do not dissociate properly, repeat step 138 and 139 until the organoid is dissociated into small clumps of 5 to 10 cells.

***Note:*** For this dissociation, it is not necessary to reach single cells. Cell clumps are enough.

141. To inactivate TrypLE, add 1 mL ADF+++ to each tube.
142. Spin the cells down at 500 × g for 5 min at 4°C.
143. Remove the supernatant with caution. At this stage, the pellet will not be visible. To be sure not to aspirate it, leave about 10–20 μL of medium on top of it.
144. Add 25 μL of the 3D-matrix to each tube and mix well.
145. Plate 25 μL of the mix in a 48-well plate (one dome per clone). Freeze the remainder of the organoid in 3D matrix in the Eppendorf tube for subsequent genotyping. Experimental procedure for this is described in step 150–156.

***Note:*** When plating the clones, we suggest keeping 5 μL of the material for genotyping (it can be stored long term at −20°C). We recommend proceeding with genotyping as soon as possible. Knowing the genotype of your clones early on will reduce the number of clones that need a split and thus reduces material costs.

146. Let the 3D-matrix solidify for 15–30 min in the incubator at 37°C.
147. In the meantime, prewarm the expansion medium supplemented with 10 μM Y-27632°C to 37°C.
148. Add expansion medium to the solidified 3D-matrix.

***Note:*** The amount of cells you get from a single organoid is limited.

149. Refresh wells every 2–3 days with expansion medium until organoids are fully grown out (Figures 6A–6C).

### Sanger sequencing based genotyping of clonally expanded organoid lines

**Timing: 1–2 days**

After clonal line generation it is key to quickly check whether you have introduced your edit of interest in your organoid line of choice. By using the leftover material from the clonal line generation steps highlighted above, Sanger sequencing validation can commence without the need to further expand the organoid clone. Unedited organoids can be kept as wild-type controls and organoids with your edit of interest can be used to assess their impact on homeostasis or disease. Excessive wild-type or edited clones may directly be discarded or stored in liquid nitrogen.

**CRITICAL:** Always take a known wild-type sequence along in your Sanger sequencing experiments to compare your edited clones to.

150. To isolate genomic DNA (gDNA) from the cell solution kept at step 145, use a commercial kit that can isolate enough gDNA starting from little material (10–100 cells) (e.g., zymogen gDNA Microprep kit) following the manufacturer’s instructions.

***Note:*** The amount of gDNA extracted from small amount of cells is so small that measuring on a nanodrop is not possible. In our experience PCR amplification according to the protocol below is possible even with unmeasurable amounts of gDNA.

***Note:*** If the PCRs described in the following steps do not work it might be needed to increase the starting gDNA material. In this scenario, we advise waiting for the growth of the organoids and to collect material later.

151. Perform PCR amplification around your specific mutation. There are different PCR master mixes available, so the quantity of reagents used can vary from kit to kit. As gDNA input is low we recommend running 40 amplification cycles to reach sufficient amounts of PCR product for subsequent Sanger validation.

***Note:*** Cycling temperature and times may need to be adapted based on the reagents used. The annealing temperature will depend on the primers and should be adjusted accordingly. Similarly, the extension step length can change based on the size of the sequence to be amplified.

152. When the PCR is finished, load 5 μL of the PCR reaction in a 1% agarose gel electrophoresis and run for 30–40 min at 120 V in TAE buffer.
153. Image gel on a Bio-Rad gel imager or any other gel imaging machine available to you.
154. If a clear single band of the expected amplicon size is observed, proceed with PCR clean-up using a commercial kit.

**Pause point:** PCR clean-up products can be stored long-term at −20°C.

***Note:*** if multiple bands appear, probably there has been unspecific amplification, see troubleshooting for possible solutions (problem 9).

***Note:*** In some cases, Sanger sequencing can show a mixed allele sequence. This implies that only one of the two alleles carries the mutation, while the other one is wild type. So, it might happen to obtain clones that are homozygous and heterozygous for the mutation of interest.

155. Perform Sanger sequencing of the amplified PCR products according to the recommendations of your local Sanger sequencing provider.
156. After Sanger sequencing traces are in, align your sequence traces in Benchling.
  a. Go to your region of interest and copy the sequence from 500 base-pairs upstream to 500 base-pairs downstream of your target mutation.
  b. Click on the **+** sign on the top left of the corner and click **DNA/RNA sequence > New DNA / RNA sequence**.
  c. In the newly opened screen, in the **“Create new”** tab name your sequence and click **Create**.
  d. An empty sequence will now open, paste the copied sequence around your target mutation here.
  e. Perform sequence alignment according to step **43.d**.
  f. Check for editing at your target location.
    i. Prime edited hepatocyte organoids (Figure 6E).
    ii. Base edited Colon organoids (Figure 6F).
    iii. Base edited endometrium organoids (Figure 6G).
157. Once Sanger sequencing is confirmed and organoids are sufficiently expanded, they are ready for downstream analysis and long-term storage.

## Expected outcomes

During the electroporation protocol, a significant amount of cell death is expected, as illustrated in Figure 4B. The GFP signal typically occurs between 14- and 18-h post-electroporation, as depicted in Figure 4D. GFP-positive cells can be isolated via sorting approximately 3–4 days after electroporation. Depending on the efficacy of the process, the proportion of sorted GFP-positive cells may range from 1% to 20%. This highly depends on the vectors you are using. In our experience, base editors and prime editors do not express very well in adult stem cells and will thus result in a lower transfection efficiency as measured by GFP positive cells. The amount of cell death, as well as the electroporation efficiency can vary between organoid type, as well as per donor. To ensure adequate outgrowth, the total count should surpass 1000 cells, although this number may be influenced by dissociation efficiency. Isogenic organoid clones can also be selected by incorporation of antibiotic resistance genes or directly by the CRISPR edit. In these cases, we perform a single cell step 4–5 days after the electroporation, with subsequent addition of the antibiotic to the culture or specific media changes for the functional selection. After around 10–14 days, clonal lines can be established by individually selecting organoids and transferring them to new wells.

We suggest to start screening for mutants by picking and clonally expanding 24 clones. A fraction of these (approximately 5%–10%) might be lost during the procedure and do not grow out. Based on our observations, most electroporation attempts yield a minimum of 2 homozygous mutant clones out of the 24 selected clones, with few instances where none of the clones contain the mutation of interest. Targeting efficiency can vary significantly across different genes and gRNAs. Base editors with NGG or NGN PAMs have shown higher efficiency for editing, particularly in C>T base changes. However, due to the excellent clonal capacity of ASC organoids these protocols enable the rapid screening of various hotspot mutations within a gene in a single round of clone picking.^51^ In addition, base editors are well-suited for multiplexing, allowing the selection of multiple mutations with the same base changes and PAMs (e.g., NGG C>T mutations) across different genes for editing.^2^

Prime editors can overcome some of the limitations of base editors by enabling gene editing in regions when designing base editing sgRNAs is not feasible, or when the target mutation includes an indel, small insertion, or deletion.

In this protocol, we show efficient editing of organoids using these techniques. However, it’s worth mentioning that next-generation CRISPR tools can also be applied to 2D cell lines or stem cells growing in 2D, such as hematopoietic stem cells or iPSCs, with similar outcome.

## Limitations

In this protocol, we describe a pipeline to use base- and prime editors to generate point mutations and knockouts without inducing DSBs like conventional CRISPR-Cas9 in ASC organoids. However, both techniques have some limitations. For instance, the creation of large insertions and deletions in genes is not possible with base editors and prime editors. Recently, new prime editors have been developed to edit larger DNA sequences.^52^

Designing sgRNAs for base editors can be challenging. A few requirements have to be met to obtain a working sgRNA. The presence of a PAM sequence adjacent to the sgRNA is crucial, but may not always be available. The editing window is restricted to a few positions (4 to 8), with positions 5 and 6 having a higher probability of successful editing compared to the others. In addition, the success rate of editing varies depending on the desired base change, with C>T conversions showing a higher likelihood of success with a NGG PAM. Although new combinations of PAM- and base conversion are constantly being developed, the current choices are still limited. This might impact significantly on your experimental design. Furthermore, base editors, like the ones we use in this study (for C>T), do not edit when there is a G in front of the C. For this reason, make sure to always be updated with the literature and find editors that do not have such a sequence context, such as CBE6b or EvoFERNY.^25^^,^^53^

Likewise, prime editors are somewhat unpredictable. To find the right combination of sgRNAs, it may be necessary to screen many guides (e.g., 100s) and this is not possible in organoids. Guides’ pre-screen can be performed in HEK293T, but this does not always reflect the editing efficiency in ASC organoids.

The delivery of Cas9 tools to organoids for isogenic line generation remains a challenge as can be seen in Figures 4 and 5. Significant levels of cell death and relatively low transfection efficiency do limit the use of these tools for screening in bulk. If only 2% of the stem cells in culture are transfected, at least 98% of the cells will not have your target edit. In this protocol, we describe the clonal steps to generate a culture where 100% of cells are edited. Yet, this is time-consuming and hard for some organoid types. Thus, delivery of next-generation CRISPR tools by lentivirus or ribonucleoprotein complexes may be a suitable alternative strategy to DNA mutations in adult stem cells.^54^^,^^55^^,^^56^

## Troubleshooting

### Problem 1

Step 39f–40: No bacterial colonies are growing on the plate after transformation.

### Potential solution

Check whether you have used the right antibiotic resistance and the right plasmid for cloning.

### Problem 2

Step 43: Sanger sequencing of ligated PE3 sgRNA or base editing vector reveals spacer sequences that correspond to your template material.

### Potential solution

This is indicative of ineffective DpnI cleavage. It is essential that this enzyme is kept cold at all times to reduce this unwanted result. Redoing the reaction with a new aliquot of DpnI should resolve the problem.

### Problem 3

Step 89: During the electroporation, the measured impedance values are too low (< 0.035 Ω) or too high (>0.055 Ω).

### Potential solution

To increase the impedance: it is needed to increase the organoid starting material.

To reduce the impedance: dilute your sample in more Opti-MEM.

### Problem 4

Step 96: Too much cell death after electroporation.

### Potential solution

The plasmid might be contaminated, remake the plasmid and make sure to purify the DNA. Try to increase the organoid starting material and do not over trypsinize the cells (15 min max). After electroporation, resuspend the cells well. This is important to get rid of the dead cell clusters which could end up in your culture and have a negative effect on your living cells. Finally, it is suggested to plate the cells really densely.

### Problem 5

Step 98: There is no GFP signal in the cells 24 h after electroporation.

### Potential solution

Either the efficiency of your electroporation is low. Or certain plasmids lead to a low GFP expression which is hard to detect by microscope or FACS. However, this does not imply your electroporation was not successful. When this happens, continue using antibiotic resistance or functional selection.

***Note:*** we suggest to always include a backup selection when you want to select with FACS sorting.

### Problem 6

Step 112: No cell death in the control during functional or antibiotic selection after 14 days.

### Potential solution

Increase the concentration of the antibiotics or small molecules for functional selection (e.g., Nutlin-3a or hygromycin-b Gold.).

### Problem 7

Step 112: All organoids are dead after antibiotic selection.

### Potential solution

Despite the introduction of a resistance cassette, some cells are intrinsically sensitive to antibiotics. Thus, it might happen that using a high concentration of antibiotics leads to cell death. We suggest to always start with a low concentration and increase it gradually after one week when cell death is not detected.

### Problem 8

Step 124: Little number of cells during FACS selection step (less than 1000).

### Potential solution

The effectiveness of outgrowth varies depending on the specific cell line and the efficiency of gRNA targeting. The goal is to plate between 1000 cells per 100 μL, with a density of 10 cells/μL. If there are not enough cells, increase the amount of starting material. Prior to sorting, ensure that the organoids have been dissociated into predominantly single cells.

### Problem 9

Step 154: There is more than one band visible in the agarose gel during the genotyping step.

### Potential solution

If the off target bands are few and faint compared to the one of interest, we would recommend to perform PCR purification. Subsequently, sequence the purified DNA using a distinct sequencing primer, which typically yields satisfactory genotyping outcomes. If this approach proves ineffective and there are only a few non-specific bands, well-separated from each other, the remaining PCR reaction can be loaded onto an agarose gel. Following this, the band of appropriate size can be cut and subjected to agarose gel purification. In cases where this method also fails or if there are numerous bands in the agarose gel making it difficult to precisely isolate the band of interest, conducting a PCR reaction with alternative primer pairs is recommended. Adjusting the PCR annealing temperature to 62°C sometimes aids in resolution. If these steps do not yield the desired results, a nested PCR reaction can be attempted.

### Problem 10

Step 156: After genotyping, Sanger sequencing results are noisy.

### Potential solution

Ensure an adequate amount of DNA is retained post-PCR purification, with a minimum concentration of 15 ng/mL, and that the DNA is eluted in nuclease free water during the PCR purification process. Utilize a sequencing primer positioned within the PCR amplification region. Consider testing an alternate sequencing primer if necessary. Additionally, organoid clones can be expanded through one or more passages to increase the quantity of genomic DNA available, thereby improving genotyping outcomes.

### Problem 11

Step 154: No homozygous mutant for the gene of interest was obtained.

### Potential solution

Another sgRNA targeting the same gene could be utilized, preferably aiming at alternative exons. The efficacy of certain sgRNAs in targeting the desired site might be diminished due to unpredictable factors such as off-target effects or closed chromatin. In situations where genes vital for cell survival are targeted, achieving effective gene knockout might be challenging, necessitating the desire for conditional genetic modifications instead.

## Resource availability

### Lead contact

Further information and requests for resources and reagents should be directed to and will be fulfilled by the lead contact, Maarten H Geurts (m.h.geurts-6@prinsesmaximacentrum.nl).

### Technical contact

For technical questions please contact Martina Celotti (m.celotti@hubrecht.eu).

### Materials availability

This study did not generate new unique reagents.

### Data and code availability

This study did not generate/analyze dataset/code.

## References

[1] Geurts M.H., Clevers H. (2023). CRISPR engineering in organoids for gene repair and disease modelling. Nat. Rev. Bioeng..

[2] Geurts M.H., Gandhi S., Boretto M.G., Akkerman N., Derks L.L.M., Van Son G., Celotti M., Harshuk-Shabso S., Peci F., Begthel H. (2023). One-step generation of tumor models by base editor multiplexing in adult stem cell-derived organoids. Nat. Commun..

[3] Kapałczyńska M., Kolenda T., Przybyła W., Zajączkowska M., Teresiak A., Filas V., Ibbs M., Bliźniak R., Łuczewski Ł., Lamperska K. (2016). 2D and 3D cell cultures – a comparison of different types of cancer cell cultures. Arch. Med. Sci..

[4] Duval K., Grover H., Han L.-H., Mou Y., Pegoraro A.F., Fredberg J., Chen Z. (2017). Modeling Physiological Events in 2D vs. 3D Cell Culture. Physiology.

[5] Jackstadt R., Sansom O.J. (2016). Mouse models of intestinal cancer. J. Pathol..

[6] Kim J., Koo B.-K., Knoblich J.A. (2020). Human organoids: model systems for human biology and medicine. Nat. Rev. Mol. Cell Biol..

[7] Clevers H. (2016). Modeling Development and Disease with Organoids. Cell.

[8] Dekkers J.F., Wiegerinck C.L., De Jonge H.R., Bronsveld I., Janssens H.M., De Winter-de Groot K.M., Brandsma A.M., De Jong N.W.M., Bijvelds M.J.C., Scholte B.J. (2013). A functional CFTR assay using primary cystic fibrosis intestinal organoids. Nat. Med..

[9] Wright A.V., Nuñez J.K., Doudna J.A. (2016). Biology and Applications of CRISPR Systems: Harnessing Nature’s Toolbox for Genome Engineering. Cell.

[10] Jinek M., Chylinski K., Fonfara I., Hauer M., Doudna J.A., Charpentier E. (2012). A Programmable Dual-RNA–Guided DNA Endonuclease in Adaptive Bacterial Immunity. Science.

[11] Leibowitz M.L., Papathanasiou S., Doerfler P.A., Blaine L.J., Sun L., Yao Y., Zhang C.-Z., Weiss M.J., Pellman D. (2021). Chromothripsis as an on-target consequence of CRISPR–Cas9 genome editing. Nat. Genet..

[12] Komor A.C., Kim Y.B., Packer M.S., Zuris J.A., Liu D.R. (2016). Programmable editing of a target base in genomic DNA without double-stranded DNA cleavage. Nature.

[13] Gaudelli N.M., Komor A.C., Rees H.A., Packer M.S., Badran A.H., Bryson D.I., Liu D.R. (2017). Programmable base editing of A·T to G·C in genomic DNA without DNA cleavage. Nature.

[14] Anzalone A.V., Randolph P.B., Davis J.R., Sousa A.A., Koblan L.W., Levy J.M., Chen P.J., Wilson C., Newby G.A., Raguram A., Liu D.R. (2019). Search-and-replace genome editing without double-strand breaks or donor DNA. Nature.

[15] Geurts M.H., De Poel E., Amatngalim G.D., Oka R., Meijers F.M., Kruisselbrink E., Van Mourik P., Berkers G., De Winter-de Groot K.M., Michel S. (2020). CRISPR-Based Adenine Editors Correct Nonsense Mutations in a Cystic Fibrosis Organoid Biobank. Cell Stem Cell.

[16] Geurts M.H., De Poel E., Pleguezuelos-Manzano C., Oka R., Carrillo L., Andersson-Rolf A., Boretto M., Brunsveld J.E., Van Boxtel R., Beekman J.M., Clevers H. (2021). Evaluating CRISPR-based prime editing for cancer modeling and CFTR repair in organoids. Life Sci. Alliance.

[17] Zafra M.P., Schatoff E.M., Katti A., Foronda M., Breinig M., Schweitzer A.Y., Simon A., Han T., Goswami S., Montgomery E. (2018). Optimized base editors enable efficient editing in cells, organoids and mice. Nat. Biotechnol..

[18] Doman J.L., Sousa A.A., Randolph P.B., Chen P.J., Liu D.R. (2022). Designing and executing prime editing experiments in mammalian cells. Nat. Protoc..

[19] Walton R.T., Christie K.A., Whittaker M.N., Kleinstiver B.P. (2020). Unconstrained genome targeting with near-PAMless engineered CRISPR-Cas9 variants. Science.

[20] Nishimasu H., Shi X., Ishiguro S., Gao L., Hirano S., Okazaki S., Noda T., Abudayyeh O.O., Gootenberg J.S., Mori H. (2018). Engineered CRISPR-Cas9 nuclease with expanded targeting space. Science.

[21] Huang T.P., Newby G.A., Liu D.R. (2021). Precision genome editing using cytosine and adenine base editors in mammalian cells. Nat. Protoc..

[22] Rees H.A., Liu D.R. (2018). Base editing: precision chemistry on the genome and transcriptome of living cells. Nat. Rev. Genet..

[23] Richter M.F., Zhao K.T., Eton E., Lapinaite A., Newby G.A., Thuronyi B.W., Wilson C., Koblan L.W., Zeng J., Bauer D.E. (2020). Phage-assisted evolution of an adenine base editor with improved Cas domain compatibility and activity. Nat. Biotechnol..

[24] Koblan L.W., Doman J.L., Wilson C., Levy J.M., Tay T., Newby G.A., Maianti J.P., Raguram A., Liu D.R. (2018). Improving cytidine and adenine base editors by expression optimization and ancestral reconstruction. Nat. Biotechnol..

[25] Thuronyi B.W., Koblan L.W., Levy J.M., Yeh W.-H., Zheng C., Newby G.A., Wilson C., Bhaumik M., Shubina-Oleinik O., Holt J.R., Liu D.R. (2019). Continuous evolution of base editors with expanded target compatibility and improved activity. Nat. Biotechnol..

[26] Zhang Z., Rong X., Xie T., Li Z., Song H., Zhen S., Wang H., Wu J., Jaffrey S.R., Li X. (2024). Fluorogenic CRISPR for genomic DNA imaging. Nat. Commun..

[27] Kurt I.C., Zhou R., Iyer S., Garcia S.P., Miller B.R., Langner L.M., Grünewald J., Joung J.K. (2021). CRISPR C-to-G base editors for inducing targeted DNA transversions in human cells. Nat. Biotechnol..

[28] Koblan L.W., Arbab M., Shen M.W., Hussmann J.A., Anzalone A.V., Doman J.L., Newby G.A., Yang D., Mok B., Replogle J.M. (2021). Efficient C·G-to-G·C base editors developed using CRISPRi screens, target-library analysis, and machine learning. Nat. Biotechnol..

[29] Tong H., Wang X., Liu Y., Liu N., Li Y., Luo J., Ma Q., Wu D., Li J., Xu C., Yang H. (2023). Programmable A-to-Y base editing by fusing an adenine base editor with an N-methylpurine DNA glycosylase. Nat. Biotechnol..

[30] Tong H., Liu N., Wei Y., Zhou Y., Li Y., Wu D., Jin M., Cui S., Li H., Li G. (2023). Programmable deaminase-free base editors for G-to-Y conversion by engineered glycosylase. Natl. Sci. Rev..

[31] Ye L., Zhao D., Li J., Wang Y., Li B., Yang Y., Hou X., Wang H., Wei Z., Liu X. (2024). Glycosylase-based base editors for efficient T-to-G and C-to-G editing in mammalian cells. Nat. Biotechnol..

[32] Drost J., Van Jaarsveld R.H., Ponsioen B., Zimberlin C., Van Boxtel R., Buijs A., Sachs N., Overmeer R.M., Offerhaus G.J., Begthel H. (2015). Sequential cancer mutations in cultured human intestinal stem cells. Nature.

[33] Lannagan T.R.M., Lee Y.K., Wang T., Roper J., Bettington M.L., Fennell L., Vrbanac L., Jonavicius L., Somashekar R., Gieniec K. (2019). Genetic editing of colonic organoids provides a molecularly distinct and orthotopic preclinical model of serrated carcinogenesis. Gut.

[34] Matano M., Date S., Shimokawa M., Takano A., Fujii M., Ohta Y., Watanabe T., Kanai T., Sato T. (2015). Modeling colorectal cancer using CRISPR-Cas9–mediated engineering of human intestinal organoids. Nat. Med..

[35] Pleguezuelos-Manzano C., Puschhof J., van den Brink S., Geurts V., Beumer J., Clevers H. (2020). Establishment and Culture of Human Intestinal Organoids Derived from Adult Stem Cells. Curr. Protoc. Immunol..

[36] Fu Y., Foden J.A., Khayter C., Maeder M.L., Reyon D., Joung J.K., Sander J.D. (2013). High-frequency off-target mutagenesis induced by CRISPR-Cas nucleases in human cells. Nat. Biotechnol..

[37] Perez-Pinera P., Kocak D.D., Vockley C.M., Adler A.F., Kabadi A.M., Polstein L.R., Thakore P.I., Glass K.A., Ousterout D.G., Leong K.W. (2013). RNA-guided gene activation by CRISPR-Cas9–based transcription factors. Nat. Methods.

[38] Nelson J.W., Randolph P.B., Shen S.P., Everette K.A., Chen P.J., Anzalone A.V., An M., Newby G.A., Chen J.C., Hsu A., Liu D.R. (2022). Engineered pegRNAs improve prime editing efficiency. Nat. Biotechnol..

[39] Doman J.L., Pandey S., Neugebauer M.E., An M., Davis J.R., Randolph P.B., McElroy A., Gao X.D., Raguram A., Richter M.F. (2023). Phage-assisted evolution and protein engineering yield compact, efficient prime editors. Cell.

[40] Kuscu C., Parlak M., Tufan T., Yang J., Szlachta K., Wei X., Mammadov R., Adli M. (2017). CRISPR-STOP: gene silencing through base-editing-induced nonsense mutations. Nat. Methods.

[41] Cunningham F., Allen J.E., Allen J., Alvarez-Jarreta J., Amode M.R., Armean I.M., Austine-Orimoloye O., Azov A.G., Barnes I., Bennett R. (2022). Ensembl 2022. Nucleic Acids Res..

[42] Hug N., Longman D., Cáceres J.F. (2016). Mechanism and regulation of the nonsense-mediated decay pathway. Nucleic Acids Res..

[43] Liu F., Huang S., Hu J., Chen X., Song Z., Dong J., Liu Y., Huang X., Wang S., Wang X., Shu W. (2023). Design of prime-editing guide RNAs with deep transfer learning. Nat. Mach. Intell..

[44] Mathis N., Allam A., Kissling L., Marquart K.F., Schmidheini L., Solari C., Balázs Z., Krauthammer M., Schwank G. (2023). Predicting prime editing efficiency and product purity by deep learning. Nat. Biotechnol..

[45] Chow R.D., Chen J.S., Shen J., Chen S. (2021). A web tool for the design of prime-editing guide RNAs. Nat. Biomed. Eng..

[46] Hsu J.Y., Grünewald J., Szalay R., Shih J., Anzalone A.V., Lam K.C., Shen M.W., Petri K., Liu D.R., Joung J.K., Pinello L. (2021). PrimeDesign software for rapid and simplified design of prime editing guide RNAs. Nat. Commun..

[47] Liu P., Liang S.-Q., Zheng C., Mintzer E., Zhao Y.G., Ponnienselvan K., Mir A., Sontheimer E.J., Gao G., Flotte T.R. (2021). Improved prime editors enable pathogenic allele correction and cancer modelling in adult mice. Nat. Commun..

[48] Nelson J.W., Randolph P.B., Shen S.P., Everette K.A., Chen P.J., Anzalone A.V., An M., Newby G.A., Chen J.C., Hsu A., Liu D.R. (2022). Engineered pegRNAs improve prime editing efficiency. Nat. Biotechnol..

[49] Tiroille V., Krug A., Bokobza E., Kahi M., Bulcaen M., Ensinck M.M., Geurts M.H., Hendriks D., Vermeulen F., Larbret F. (2023). Nanoblades allow high-level genome editing in murine and human organoids. Mol. Ther. Nucleic Acids.

[50] Sun D., Evans L., Perrone F., Sokleva V., Lim K., Rezakhani S., Lutolf M., Zilbauer M., Rawlins E.L. (2021). A functional genetic toolbox for human tissue-derived organoids. Elife.

[51] Boretto M., Geurts M.H., Gandhi S., Ma Z., Staliarova N., Celotti M., Lim S., He G.-W., Millen R., Driehuis E. (2024). Epidermal growth factor receptor (EGFR) is a target of the tumor-suppressor E3 ligase FBXW7. Proc. Natl. Acad. Sci..

[52] Anzalone A.V., Gao X.D., Podracky C.J., Nelson A.T., Koblan L.W., Raguram A., Levy J.M., Mercer J.A.M., Liu D.R. (2022). Programmable deletion, replacement, integration and inversion of large DNA sequences with twin prime editing. Nat. Biotechnol..

[53] Zhang E., Neugebauer M.E., Krasnow N.A., Liu D.R. (2024). Phage-assisted evolution of highly active cytosine base editors with enhanced selectivity and minimal sequence context preference. Nat. Commun..

[54] Petri K., Zhang W., Ma J., Schmidts A., Lee H., Horng J.E., Kim D.Y., Kurt I.C., Clement K., Hsu J.Y. (2022). CRISPR prime editing with ribonucleoprotein complexes in zebrafish and primary human cells. Nat. Biotechnol..

[55] Lin S.-C., Haga K., Zeng X.-L., Estes M.K. (2022). Generation of CRISPR–Cas9-mediated genetic knockout human intestinal tissue–derived enteroid lines by lentivirus transduction and single-cell cloning. Nat. Protoc..

[56] Platt R.J., Chen S., Zhou Y., Yim M.J., Swiech L., Kempton H.R., Dahlman J.E., Parnas O., Eisenhaure T.M., Jovanovic M. (2014). CRISPR-Cas9 Knockin Mice for Genome Editing and Cancer Modeling. Cell.

